# ERα–PADI4–CXCL8 Axis Drives MDSC‐Mediated Immune Evasion in Anaplastic Thyroid Carcinoma

**DOI:** 10.1002/advs.77901

**Published:** 2026-10-22

**Authors:** Dongdong Zhang, Ying Cai, Junbin Zhang, Jianyong Zhang, Ran Jing, Ming Cai

**Affiliations:** ^1^ Department of Thyroid Oncology Chongqing Key Laboratory of Translational Research For Cancer Metastasis and Individualized Treatment Chongqing University Cancer Hospital Chongqing People's Republic of China

**Keywords:** anaplastic thyroid carcinoma, CXCL8, estrogen receptor alpha, histone citrullination, myeloid‐derived suppressor cells, PADI4

## Abstract

Anaplastic thyroid carcinoma (ATC) is a highly lethal malignancy with a median survival of merely 4–6 months and no effective systemic therapies. Although ATC predominantly arises in elderly postmenopausal women with negligible circulating 17β‐estradiol (E2), we observed that estrogen receptor alpha (ERα) is constitutively phosphorylated at serine 118 (S118) in ATC tissues, independently of its canonical ligand. Here, we identify peptidyl arginine deiminase 4 (PADI4) as a direct transcriptional target of ligand‐independently activated ERα, driven by EGF/MAPK signaling. PADI4 is overexpressed in ATC along a dedifferentiation gradient, and its high expression defines an immune‐excluded tumor microenvironment characterized by dense CD33^+^ myeloid‐derived suppressor cell (MDSC) infiltration and CD8^+^ T cell exclusion, portending poor prognosis. Mechanistically, PADI4 citrullinates histone H3 at the CXCL8 promoter, opening chromatin and driving CXCL8 secretion, which recruits MDSCs through CXCR2. Pharmacological inhibition of PADI4 with GSK484 triggers compensatory CXCL1 upregulation, sustaining residual MDSC chemotaxis through CXCR1/2. Combined blockade of PADI4 (GSK484) and CXCR1/2 (SX‐682) synergistically suppresses tumor growth, eliminates pulmonary metastasis, and achieves 80% long‐term survival in an orthotopic ATC model, without appreciable systemic toxicity. These findings establish the ERα–PADI4–CXCL8–MDSC signaling axis as a central driver of immune evasion in ATC and provide a preclinical rationale for dual‐targeted epigenetic‐immunotherapy.

## Graphical abstract

MAPK‐driven ligand‐independent ERα activation drives PADI4 transcription and translation. This enzyme then modifies CXCL8 chemokines through citrullination, generating a potent chemoattractant gradient specifically for myeloid‐derived suppressor cells. Their subsequent accumulation creates a formidable immunosuppressive shield that effectively blocks cytotoxic CD8^+^ T cell infiltration. This signaling cascade unveils a promising target for reactivating suppressed anti‐tumor immunity.

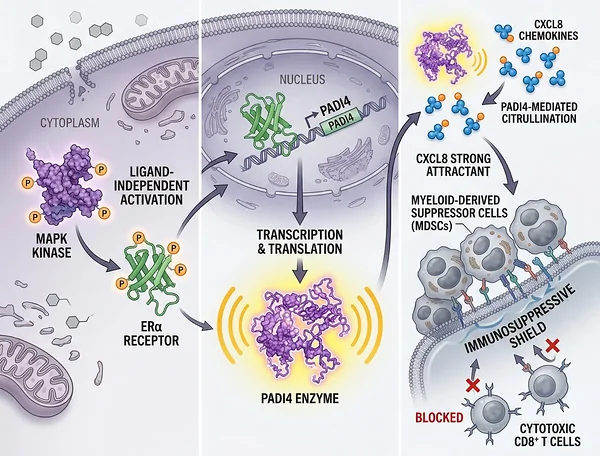

## 1 Introduction

Thyroid cancer is the most prevalent endocrine malignancy, with its global incidence having risen substantially over the past four decades [1]. While the vast majority of differentiated thyroid cancers—particularly papillary thyroid carcinoma (PTC)—carry an excellent prognosis following standard treatment with surgery, radioactive iodine, and thyroid‐stimulating hormone suppression, a small subset of patients develops highly aggressive, undifferentiated disease [2]. Anaplastic thyroid carcinoma (ATC) represents the most lethal form of thyroid cancer: although it accounts for less than 2% of all thyroid malignancies, it is responsible for a disproportionately high fraction of thyroid cancer‐related mortality [3]. The median overall survival for ATC patients remains merely 4 to 6 months, and fewer than 20% of patients survive beyond 1 year from diagnosis, a statistic that has remained essentially unchanged despite decades of clinical research [4]. The molecular landscape of ATC is characterized by extreme genomic complexity, with frequent alterations in the MAPK and PI3K‐AKT‐mTOR signaling cascades, coupled with a profoundly immunosuppressive tumor microenvironment (TME) that renders immune checkpoint inhibitors largely ineffective [5].

Among the oncogenic drivers of ATC, dysregulated kinase signaling—particularly hyperactivation of the mitogen‐activated protein kinase (MAPK) pathway through BRAF V600E, RAS mutations, or receptor tyrosine kinase amplification—is a near‐universal feature [6]. While this has prompted the clinical evaluation of targeted therapies such as dabrafenib plus trametinib for BRAF‐mutated ATC, the majority of patients lack actionable mutations and inevitably develop therapeutic resistance [7]. Intriguingly, mounting evidence indicates that ATC predominantly arises in elderly, postmenopausal women—a demographic characterized by profoundly low circulating E2 levels [8]. This clinical observation presents a paradox that has remained largely unexplored: if estrogen receptor alpha (ERα) signaling is functionally relevant in ATC, how is it sustained in the near‐absence of its canonical ligand? A critical clue emerges from the well‐established phenomenon of ligand‐independent ERα activation. In breast cancer, extensive research has demonstrated that growth factor‐activated MAPK can directly phosphorylate ERα at serine 118 (S118), triggering its nuclear translocation and transcriptional activity independently of E2 [9]. This non‐genomic mechanism enables ERα to function as a downstream effector of oncogenic kinase cascades, driving the transcription of target genes that promote proliferation and survival. However, whether this paradigm extends to ATC—and which downstream transcriptional targets ERα engages in this context—has not been systematically investigated.

A particularly attractive candidate ERα target gene in ATC is peptidyl arginine deiminase 4 (PADI4). PADI4 is a calcium‐dependent enzyme that catalyzes the post‐translational conversion of peptidyl‐arginine to peptidyl‐citrulline, a process known as citrullination, which fundamentally alters protein charge, structure, and function [10]. Unlike other members of the PADI family, PADI4 uniquely contains a nuclear localization signal and functions as both a histone modifier and a transcriptional co‐regulator. By citrullinating histone H3 at arginine residues 2, 8, 17, and 26, PADI4 can loosen chromatin compaction and regulate the accessibility of gene promoters to the transcriptional machinery [11]. Emerging evidence has implicated PADI4 in the pathogenesis of multiple malignancies: in gastric cancer, PADI4 promotes epithelial‐mesenchymal transition and metastasis through the upregulation of interleukin‐8 (CXCL8) [12]; PADI4‐mediated histone hypercitrullination has been implicated in chromatin decondensation and is associated with aggressive tumor phenotypes [13]; and in acute myeloid leukemia, PADI4 maintains leukemia stem cell self‐renewal through epigenetic reprogramming [14]. Critically, recent studies have revealed a dual role for PADI4 in tumor immunity. On one hand, PADI4 functions as a tumor suppressor in a p53‐dependent manner, with an intact immune system required for its tumor‐suppressive activity [15]. On the other hand, PADI4 negatively regulates anti‐tumor immunity by citrullinating STAT1 and thereby attenuating interferon‐γ signaling and MHC‐II expression in tumor‐associated macrophages [16]. Furthermore, myeloid‐specific deletion of PADI4 has been shown to reduce the immunosuppressive function of polymorphonuclear myeloid‐derived suppressor cells (PMN‐MDSCs), leading to enhanced T cell‐mediated tumor rejection in multiple syngeneic models [17].

The connection between PADI4 and MDSC biology is of particular relevance to ATC. MDSCs are a heterogeneous population of pathologically activated immature myeloid cells that accumulate in the TME and potently suppress both innate and adaptive anti‐tumor immune responses [18]. Their recruitment to tumors is orchestrated by a network of chemokine‐receptor axes, among which the CXCL8‐CXCR1/2 and CXCL1/2/5‐CXCR2 pathways are paramount [19]. Elevated serum CXCL8 levels and intratumoral MDSC infiltration have been correlated with advanced disease stage, resistance to immunotherapy, and poor prognosis across a spectrum of solid tumors [19]. In the context of ATC, the immune landscape is dominated by robust MDSC infiltration with concomitant exclusion of CD8^+^ cytotoxic T lymphocytes, creating an immunologically “cold” tumor that is refractory to checkpoint blockade [20]. Whether PADI4 contributes to this immune‐excluded phenotype by transcriptionally regulating chemokine expression in ATC cells has not been determined.

The convergence of these seemingly disparate observations—MAPK‐driven, ligand‐independent ERα activation; PADI4‐mediated epigenetic regulation of chemokine transcription; and MDSC‐dependent immune evasion—raises a compelling but untested hypothesis: the ERα‐PADI4 axis may function as a previously unrecognized signaling unit that integrates oncogenic kinase activity with the orchestration of an immunosuppressive TME in ATC. Specifically, we hypothesized that in the low‐estrogen environment of elderly, postmenopausal ATC patients, ERα is constitutively phosphorylated at S118 by the MAPK pathway, leading to direct transcriptional activation of PADI4. In turn, PADI4 citrullinates histones at chemokine gene promoters—particularly CXCL8—to open chromatin and facilitate their transcription, driving the secretion of chemokines that recruit MDSCs into the tumor bed. To test this hypothesis, we assembled a multi‐cohort clinical dataset to examine the prognostic significance and immune correlates of PADI4 expression in ATC, performed a series of in vitro and in vivo functional experiments to dissect the upstream and downstream regulatory mechanisms, and evaluated a dual‐targeted therapeutic strategy combining a PADI4 inhibitor with a CXCR1/2 antagonist. Our results establish the ERα‐PADI4‐CXCL8‐MDSC signaling axis as a critical driver of immune evasion and tumor progression in ATC and identify actionable therapeutic vulnerabilities within this pathway.

## 2 Materials and Methods

### 2.1 Human Tissue Samples and Serum Collection

Multi‐cohort transcriptomic dataset. PADI4 and ESR1 mRNA expression data were compiled from three sources: (1) RNA‐seq data from normal thyroid tissues (n = 59) obtained from the Genotype‐Tissue Expression (GTEx) project; (2) RNA‐seq data from PTC tissues (n = 365) obtained from The Cancer Genome Atlas (TCGA) THCA cohort; and (3) in‐house RNA‐seq data from pathologically confirmed PDTC (n = 28) and ATC (n = 65) specimens collected at Chongqing University Cancer Hospital between January 2018 and December 2023. All in‐house specimens were snap‐frozen in liquid nitrogen within 15 min of surgical resection and stored at −80°C until RNA extraction.

Fresh‐frozen tissue cohort for protein analysis. Fresh‐frozen thyroid tissue specimens (normal thyroid, n = 6; PTC, n = 6; ATC, n = 8) were obtained from an independent set of patients undergoing thyroidectomy during the same period. Tissues were snap‐frozen in liquid nitrogen within 15 min of resection and stored at −80°C until protein extraction.

Tissue microarray cohort for IHC and survival analysis. Formalin‐fixed, paraffin‐embedded (FFPE) tissue blocks from a third independent cohort of 78 ATC patients, 40 PTC patients, and 20 individuals with normal thyroid (from contralateral lobes of benign tumors) were used for construction of tissue microarrays (TMAs). For each case, three representative 1.0 mm cores were punched from donor blocks and arrayed into recipient blocks using a manual tissue arrayer (Beecher Instruments). Additionally, 4 µm serial sections were cut from whole FFPE blocks of ATC tissues for multiplex immunofluorescence. For p‐ERα(S118) IHC, 65 of the 78 ATC cases on the TMA had sufficient residual tumor tissue after completion of PADI4 staining and multiplex immunofluorescence cycles to allow additional serial sections for phospho‐specific IHC. The 13 excluded cases were excluded solely on the basis of tissue exhaustion. Review of their clinical records confirmed that these cases were randomly distributed with respect to PADI4 H‐score, age, sex, and TNM stage, and their exclusion did not introduce systematic selection bias. Pre‐operative serum samples were collected from young PTC patients (<45 years, n = 25) and elderly ATC patients (≥60 years, n = 30). All serum samples were aliquoted and stored at −80°C until estradiol measurement. Clinicopathological data were retrieved from electronic medical records and reviewed by two independent pathologists. The study was approved by the Ethics Committee of Chongqing University Cancer Hospital (approval number: 2023‐0818), and written informed consent was obtained from all participants in accordance with the Declaration of Helsinki.

### 2.2 Cell Lines and Culture Conditions

The human ATC cell lines 8505C, CAL‐62, BHT‐101, KTC‐1, along with the monocytic leukemia cell line THP‐1, were obtained from the American Type Culture Collection (ATCC) or the Japanese Collection of Research Bioresources (JCRB) Cell Bank. All ATC cell lines were authenticated by short tandem repeat (STR) profiling within 6 months of use and tested negative for mycoplasma contamination using the MycoAlert Mycoplasma Detection Kit (Lonza). 8505C, CAL‐62, and BHT‐101 cells were cultured in RPMI‐1640 medium (Gibco) supplemented with 10% fetal bovine serum (FBS, Gibco), 100 U/mL penicillin, and 100 µg/mL streptomycin. KTC‐1 cells were maintained in DMEM/F12 (1:1) medium with 10% FBS and antibiotics. For experiments requiring estrogen‐depleted conditions, cells were cultured in phenol red‐free RPMI‐1640 (Gibco) supplemented with 10% charcoal‐stripped FBS (CSS, Biological Industries) for at least 48 h prior to stimulation. All cells were maintained at 37°C in a humidified atmosphere containing 5% CO_2_.

### 2.3 RNA Sequencing and Bioinformatic Analysis

8505C cells stably transduced with sh‐NC or sh‐PADI4 #1 lentivirus were harvested after 72 h of puromycin selection (2 µg/mL). Total RNA was extracted using the RNeasy Mini Kit (Qiagen) with on‐column DNase I digestion. RNA integrity number (RIN) was assessed using the Agilent 2100 Bioanalyzer; all samples had RIN > 8.0. Library preparation was performed using the TruSeq Stranded mRNA Library Prep Kit (Illumina) with 1 µg of total RNA as input. Libraries were sequenced on an Illumina NovaSeq 6000 platform (150 bp paired‐end reads) at a depth of approximately 40 million reads per sample. Raw reads were trimmed using Trimmomatic (v0.39) to remove adapters and low‐quality bases. Trimmed reads were aligned to the human reference genome GRCh38 using STAR (v2.7.9a) with default parameters. Gene‐level read counts were quantified using featureCounts (v2.0.1). Differential expression analysis was performed with DESeq2 (v1.34.0) in R (v4.1.2). Genes with |log_2_ fold change| > 1.0 and Benjamini‐Hochberg adjusted *p*‐value < 0.05 were considered significantly differentially expressed. For heatmap visualization, regularized log‐transformed (rlog) counts were row Z‐score normalized. Hierarchical clustering was performed using Euclidean distance and complete linkage. Volcano plots were generated using the EnhancedVolcano package (v1.12.0). KEGG pathway enrichment analysis was performed using clusterProfiler (v4.2.2). The RNA‐seq data that support the findings of this study are available from the corresponding author upon reasonable request. Access to individual‐level data may be subject to restrictions in accordance with institutional ethics policies and participant consent agreements. To minimize potential batch effects arising from the integration of transcriptomic data from different sources, the ComBat‐seq algorithm (sva package, R/Bioconductor) was applied to raw count data, with data source (GTEx, TCGA, or in‐house) specified as the batch variable. Batch‐corrected expression values were used for all cross‐cohort comparisons. To adjust for potential confounding by clinical variables, multivariate linear regression was performed with PADI4 expression as the dependent variable and histological group, age, sex, and data source as independent variables. After adjustment for all covariates, histological group remained the dominant determinant of PADI4 expression (β for ATC vs. Normal = 5.62, 95% CI: 4.85–6.39, *p <* 0.001; Table S1).

### 2.4 TCGA Pan‐Cancer Analysis

Pan‐cancer RNA‐seq data (log_2_ TPM) and corresponding clinical annotations were downloaded from the UCSC Xena browser (https://xena.ucsc.edu/) for the TCGA TARGET GTEx dataset. Spearman correlation coefficients between ESR1 and PADI4 mRNA expression were calculated for each cancer type. Data visualization was performed using the ggpubr package in R. The pan‐cancer scatter plot with a fitted linear regression line represents log_2_‐transformed TPM values across approximately 500 samples from more than 30 tumor types.

### 2.5 Quantitative Real‐Time PCR (qPCR)

Total RNA was reverse‐transcribed into cDNA using the PrimeScript RT Master Mix (Takara). qPCR was performed using TB Green Premix Ex Taq II (Takara) on a QuantStudio 6 Flex Real‐Time PCR System (Applied Biosystems). The thermal cycling conditions were: 95°C for 30 s, followed by 40 cycles of 95°C for 5 s and 60°C for 30 s. A dissociation curve analysis was performed at the end of each run to confirm amplification specificity. Relative gene expression was calculated using the 2^(‐ΔΔCt) method with GAPDH as the internal reference. All reactions were performed in quadruplicate, and at least three independent biological replicates were analyzed.

### 2.6 Western Blot Analysis

Cells or homogenized tissue samples were lysed in RIPA lysis buffer (Beyotime) supplemented with protease inhibitor cocktail (Roche), phosphatase inhibitor cocktail (Roche), and 1 mM phenylmethylsulfonyl fluoride (PMSF). Protein concentration was determined using the BCA Protein Assay Kit (Beyotime). Equal amounts of protein (30 µg per lane) were separated by 10% SDS‐PAGE and transferred onto polyvinylidene difluoride (PVDF) membranes (Millipore). Membranes were blocked with 5% (w/v) bovine serum albumin (BSA) in Tris‐buffered saline containing 0.1% Tween‐20 (TBST) for 1 h at room temperature, and then incubated with primary antibodies at 4°C overnight. The following primary antibodies were used: anti‐p‐ERα (S118) (rabbit monoclonal, CST #2511, 1:1000), anti‐ERα (rabbit monoclonal, CST #8644, 1:1000), anti‐PADI4 (rabbit monoclonal, Abcam ab214810, 1:1000), anti‐p‐ERK1/2 (Thr202/Tyr204) (rabbit monoclonal, CST #4370, 1:1000), anti‐ERK1/2 (rabbit monoclonal, CST #4695, 1:1000), and anti‐GAPDH (rabbit monoclonal, CST #5174, 1:5000). After three washes with TBST, membranes were incubated with horseradish peroxidase (HRP)‐conjugated goat anti‐rabbit IgG secondary antibody (CST #7074, 1:5000) for 1 h at room temperature. Protein bands were visualized using SuperSignal West Pico Chemiluminescent Substrate (Thermo Fisher) on a ChemiDoc XRS+ imaging system (Bio‐Rad). Band intensities were quantified using ImageJ software (NIH, v1.53t) and normalized to GAPDH.

### 2.7 Immunohistochemistry (IHC)

TMA sections (4 µm) were deparaffinized in xylene, rehydrated through graded ethanol, and subjected to heat‐induced antigen retrieval in citrate buffer (pH 6.0) using a microwave oven for 15 min. Endogenous peroxidase activity was blocked with 3% H_2_O_2_ for 15 min at room temperature. Sections were incubated with anti‐PADI4 primary antibody (Abcam ab214810, 1:200), anti‐Ki67 antibody (CST #9449, 1:400), or anti‐cleaved caspase‐3 (Asp175) antibody (CST #9661, 1:400) at 4°C overnight, followed by HRP‐conjugated secondary antibody (EnVision+ System, Dako) for 30 min at room temperature. Diaminobenzidine (DAB) was used as the chromogen, and sections were counterstained with hematoxylin. For IHC scoring, the H‐score was calculated as the product of staining intensity (0, negative; 1, weak; 2, moderate; 3, strong) and the percentage of positively stained cells (0%–100%), producing a range of 0–300. Scoring was performed independently by two board‐certified pathologists blinded to clinical data.

### 2.8 Multiplex Immunofluorescence (mIF)

Four‐micrometer FFPE sections of ATC tumors were subjected to multiplex immunofluorescence staining using the Opal 7‐Color Manual IHC Kit (Akoya Biosciences, NEL811001KT). The staining protocol involved iterative cycles of heat‐induced antigen retrieval, primary antibody incubation, HRP‐conjugated secondary antibody, and Opal fluorophore tyramide signal amplification, with a microwave‐based antibody stripping step between each cycle. The following antibodies and Opal fluorophores were used in sequence: anti‐PADI4 (Abcam ab214810, 1:200) with Opal 570 (red), anti‐CD33 (clone HIM3‐4, BioLegend 366610, 1:100) with Opal 520 (green), anti‐CD8 (clone C8/144B, Dako M7103, 1:100) with Opal 690 (cyan). DAPI was used for nuclear counterstaining. Slides were mounted with ProLong Gold Antifade Mountant (Thermo Fisher) and imaged on a Leica TCS SP8 confocal laser scanning microscope using a 40× or 63× oil immersion objective. For quantitative analysis, at least five representative high‐power fields per specimen were captured and analyzed using HALO image analysis software (Indica Labs, v3.4). PADI4‐high and PADI4‐low regions were defined as areas with the top 30% and bottom 30% of PADI4 fluorescence intensity, respectively. Cell densities (cells/mm^2^) of CD33^+^ MDSCs and CD8^+^ T cells were quantified within each region.

### 2.9 Confocal Immunofluorescence

8505C cells were seeded on glass coverslips, serum‐starved for 16 h, and stimulated with EGF (50 ng/mL) for 30 min. Cells were fixed with 4% paraformaldehyde for 15 min, permeabilized with 0.2% Triton X‐100 for 10 min, and blocked with 5% BSA for 1 h at room temperature. Coverslips were incubated with anti‐ERα (CST #8644, 1:200) and anti‐PADI4 (Abcam ab214810, 1:100) primary antibodies at 4°C overnight, followed by incubation with Alexa Fluor 488‐conjugated anti‐rabbit IgG and Alexa Fluor 555‐conjugated anti‐mouse IgG secondary antibodies (Thermo Fisher, 1:500) for 1 h at room temperature in the dark. Coverslips were mounted with ProLong Gold Antifade Mountant containing DAPI (Thermo Fisher). Images were acquired on a Leica TCS SP8 confocal laser scanning microscope using a 63× oil immersion objective (NA 1.4). Sequential channel acquisition was used to avoid spectral bleed‐through. For each experimental condition, at least 10 fields were captured, and images were processed using Leica LAS X software and ImageJ (NIH, v1.53t).

### 2.10 Plasmid Constructs, shRNA, siRNA, and Lentivirus Production

The full‐length human PADI4 coding sequence (NM_012387.2) was PCR‐amplified from 8505C cDNA and cloned into the pCDH‐CMV‐MCS‐EF1‐Puro lentiviral vector (System Biosciences) with a C‐terminal FLAG tag. The catalytically inactive C645S mutant was generated by site‐directed mutagenesis using the QuikChange Lightning Kit (Agilent) and verified by Sanger sequencing. For ERα overexpression, the human ESR1 coding sequence was cloned into the same lentiviral backbone. Two independent shRNAs targeting human PADI4 (sh‐PADI4 #1: 5′‐GCCAGTGTTCTACAGCTATAA‐3′; sh‐PADI4 #2: 5′‐GCAGATCAACCAGTGTTCTTA‐3′) and a non‐targeting control shRNA (sh‐NC: 5′‐CCTAAGGTTAAGTCGCCCTCG‐3′) were cloned into the pLKO.1‐puro vector (Addgene #8453). For ERα knockdown, ON‐TARGETplus SMARTpool siRNA targeting human ESR1 (L‐003401‐00) and a non‐targeting control siRNA (D‐001810‐10) were purchased from Dharmacon. Lentiviruses were produced by co‐transfecting 293T cells with the transfer plasmid, psPAX2 (Addgene #12260), and pMD2.G (Addgene #12259) using Lipofectamine 3000 (Thermo Fisher). Viral supernatants were collected at 48 and 72 h post‐transfection, filtered through 0.45 µm membranes, and concentrated using Lenti‐X Concentrator (Takara). Cells were transduced in the presence of 8 µg/mL polybrene and selected with 2 µg/mL puromycin for 72 h.

### 2.11 Cell Proliferation, Colony Formation, and Apoptosis Assays

Cell proliferation was assessed using the Cell Counting Kit‐8 (CCK‐8, Dojindo). Briefly, 2000 cells per well were seeded in 96‐well plates, and 10 µL of CCK‐8 reagent was added at each time point (Days 0, 1, 2, 3, 4, and 5). After incubation at 37°C for 2 h, absorbance was measured at 450 nm using a microplate reader (BioTek). For EdU incorporation assays, cells were incubated with 10 µM EdU for 2 h, fixed, permeabilized, and detected using the Click‐iT EdU Alexa Fluor 594 Imaging Kit (Thermo Fisher) following the manufacturer's instructions. Nuclei were counterstained with Hoechst 33342. Images were acquired on a Leica DMi8 fluorescence microscope, and the percentage of EdU‐positive cells was calculated from five random fields per sample. For colony formation assays, 500 cells per well were seeded in 6‐well plates and cultured for 14 days with medium changes every 3 days. Colonies were fixed with methanol, stained with 0.1% crystal violet, and photographed. Colonies containing more than 50 cells were counted manually. For apoptosis analysis, cells were harvested after 24 h of serum starvation, stained with Annexin V‐FITC and propidium iodide (PI) using the FITC Annexin V Apoptosis Detection Kit (BD Biosciences), and analyzed on a BD FACSCanto II flow cytometer. Data were processed using FlowJo software (v10.8.1).

### 2.12 Migration, Invasion, and Wound Healing Assays

For Transwell migration assays, 5 × 10^4^ cells in 200 µL of serum‐free RPMI‐1640 were seeded into the upper chamber of a Transwell insert (Corning, 8 µm pore size). The lower chamber was filled with 700 µL of RPMI‐1640 containing 20% FBS as a chemoattractant. After 24 h, non‐migrated cells on the upper surface of the membrane were removed with a cotton swab. Migrated cells on the lower surface were fixed with methanol, stained with 0.1% crystal violet, and photographed under a light microscope. Cells were counted in five random fields per insert. For invasion assays, the same procedure was followed except that the upper chamber was pre‐coated with 50 µL of Matrigel (Corning, 1:8 dilution in serum‐free RPMI‐1640) and allowed to polymerize at 37°C for 2 h prior to cell seeding. For wound healing assays, cells were seeded in 6‐well plates and grown to confluence. A straight scratch wound was created using a 200 µL sterile pipette tip. Cell debris was removed by washing with PBS, and the monolayer was incubated in serum‐free RPMI‐1640. Phase‐contrast images of the wound area were captured at 0 and 24 h using an inverted microscope. Wound closure was calculated as [(wound width at 0 h − wound width at 24 h) / wound width at 0 h] × 100%.

### 2.13 Chromatin Immunoprecipitation (ChIP‐qPCR)

ChIP assays were performed using the SimpleChIP Plus Enzymatic Chromatin IP Kit (CST #9005). Briefly, 4 × 10^6^ 8505C cells were crosslinked with 1% formaldehyde for 10 min at room temperature, followed by quenching with 125 mM glycine for 5 min. Nuclei were isolated and chromatin was digested with micrococcal nuclease to obtain fragments of 150–900 bp. For H3Cit ChIP, 15 µg of digested chromatin was incubated with 5 µg of anti‐Histone H3 (citrulline R2 + R8 + R17) antibody (Abcam ab219407) or normal rabbit IgG as a negative control at 4°C overnight with rotation. For ERα ChIP, anti‐ERα antibody (CST #8644, 5 µg) was used. Immune complexes were captured with Protein G magnetic beads, washed sequentially with low‐salt and high‐salt wash buffers, and eluted. Crosslinks were reversed by incubation with proteinase K at 65°C for 2 h. DNA was purified using spin columns and analyzed by qPCR with primers targeting the CXCL8 promoter region (−300 to −50 bp from the transcription start site) containing the known citrullination peak, or the PADI4 promoter region (−1500 to −1200 bp) containing the predicted ERE motif. The percentage of input was calculated as 2^(Ct_input − 6.644 − Ct_ChIP) × 100%.

### 2.14 ATAC‐qPCR

ATAC‐qPCR was performed as previously described with modifications. Briefly, 5 × 10^4^ cells were washed with cold PBS, lysed in cold lysis buffer (10 mM Tris‐HCl, pH 7.4, 10 mM NaCl, 3 mM MgCl_2_, 0.1% NP‐40), and centrifuged at 500 g for 10 min to pellet nuclei. Nuclei were resuspended in a transposase reaction mix containing Tn5 transposase (Illumina Tagment DNA Enzyme) and incubated at 37°C for 30 min. Tagmented DNA was purified using the MinElute PCR Purification Kit (Qiagen). qPCR was performed using primers flanking the CXCL8 promoter region (the same region assessed by ChIP‐qPCR) and a negative control intergenic region (chr12: 53 456 789–53 456 988, GRCh38/hg38). Data were normalized to mitochondrial DNA copy number, and fold enrichment relative to the negative control region was calculated using the ΔΔCt method.

### 2.15 Dual‐Luciferase Reporter Assay

The human CXCL8 promoter fragment (−500 to +50 bp relative to the transcription start site) was PCR‐amplified from human genomic DNA and cloned into the pGL3‐Basic firefly luciferase reporter vector (Promega) using KpnI and HindIII restriction sites. For the PADI4 promoter dual‐luciferase assay, a similar strategy was employed to clone the PADI4 promoter region (−1500 to −1200 bp containing the wild‐type ERE motif). The ERE‐mutant PADI4 promoter was generated by site‐directed mutagenesis, changing the core ERE sequence from AGGTCAnnnTGACCT to AGATCTnnnTCTAGA. All constructs were verified by Sanger sequencing. For reporter assays, 8505C cells or 293T cells were seeded in 24‐well plates and co‐transfected with 400 ng of firefly luciferase reporter plasmid, 10 ng of pRL‐TK Renilla luciferase internal control plasmid, and 400 ng of effector plasmid (pCDNA3.1‐Vector, pCDNA3.1‐WT‐PADI4, pCDNA3.1‐C645S‐PADI4, or pCDH‐ERα‐OE) using Lipofectamine 3000. After 48 h, cells were lysed, and firefly and Renilla luciferase activities were measured using the Dual‐Glo Luciferase Assay System (Promega) on a GloMax 20/20 luminometer (Promega). Relative luciferase activity was calculated as the ratio of firefly to Renilla luminescence.

### 2.16 MDSC Isolation and Culture

Mouse bone marrow‐derived MDSCs were isolated from 8‐ to 12‐week‐old female BALB/c mice. Briefly, bone marrow cells were flushed from femoral and tibial cavities with RPMI‐1640 medium. After red blood cell lysis, CD11b^+^Gr‐1^+^ MDSCs were enriched using magnetic bead‐conjugated antibodies and a magnetic cell sorting system (MACS, Miltenyi Biotec). Purity exceeded 90% as confirmed by flow cytometry.

### 2.17 MDSC Chemotaxis Transwell Assay

For MDSC chemotaxis assays, conditioned medium (CM) from 8505C cells stably transduced with sh‐NC or sh‐PADI4 was collected after 48 h of culture in serum‐free RPMI‐1640 and centrifuged at 1000 g for 10 min to remove debris. CM (700 µL) was placed in the lower chamber of a Transwell insert (8 µm pore size). Freshly isolated mouse MDSCs (5 × 10^4^ cells in 100 µL serum‐free RPMI‐1640) were seeded into the upper chamber. For rescue experiments, recombinant human CXCL8 (rCXCL8, PeproTech, 50 ng/mL) was added to the lower chamber CM. For blocking experiments, MDSCs were pre‐incubated for 30 min with 10 µg/mL anti‐CXCR2 neutralizing antibody (clone 48311, R&D Systems) or control IgG before seeding. For experiments evaluating GSK484 (MedChemExpress) treatment effects, 8505C cells were treated with DMSO or GSK484 (10 µM) for 24 h prior to CM collection. After 6 h of incubation at 37°C, migrated MDSCs on the lower surface of the membrane were fixed, stained, and counted as described for the Transwell migration assay above.

### 2.18 CFSE‐Based T Cell Proliferation Assay

CD8^+^ T cells were isolated from splenocytes of BALB/c mice using the EasySep Mouse CD8^+^ T Cell Isolation Kit (STEMCELL Technologies). Purified CD8^+^ T cells were labeled with 5 µM carboxyfluorescein succinimidyl ester (CFSE, Thermo Fisher) and activated with anti‐CD3/CD28 beads (Dynabeads, Thermo Fisher) at a 5:1 bead‐to‐cell ratio. T cells (5 × 10^4^ per well) were co‐cultured with sh‐NC‐educated or sh‐PADI4‐educated MDSCs at a T:MDSC ratio of 5:1 in RPMI‐1640 medium containing 10% FBS and 30 IU/mL recombinant human IL‐2 (PeproTech). T cells cultured alone (without MDSCs) served as a positive control for proliferation. After 72 h, cells were collected and analyzed by flow cytometry using a BD FACSCanto II flow cytometer. CFSE fluorescence intensity was measured in the FITC channel. The number of cell divisions was inferred from the progressive halving of CFSE fluorescence intensity, with each successive peak representing one additional round of division. Data were analyzed using the Proliferation Modeling module of FlowJo software (v10.8.1).

### 2.19 In Vivo Tumor Models

All animal experiments were approved by the Institutional Animal Care and Use Committee (IACUC) of Chongqing University Cancer Hospital (approval number: 2023‐0858) and conducted in accordance with institutional and national guidelines. Female BALB/c nude mice (6–8 weeks old, 18–22 g) were purchased from Charles River Laboratories and housed under specific pathogen‐free (SPF) conditions with a 12 h light/dark cycle and ad libitum access to autoclaved food and water.

For subcutaneous tumor models, 8505C cells stably expressing sh‐NC or sh‐PADI4 (1 × 10^6^ cells in 100 µL of PBS mixed with 100 µL of Matrigel) were injected subcutaneously into the right flank of BALB/c nude mice (n = 8 per group). Tumor length (L) and width (W) were measured every 3 days using digital calipers, and tumor volume was calculated using the standard ellipsoid approximation formula: V = L × W ^2^/ 2. Mice were euthanized on Day 30, and tumors were excised, weighed, and photographed. Tumor tissues were divided into two parts: one part was fixed in 10% neutral buffered formalin for paraffin embedding and histological analysis, and the other part was snap‐frozen in liquid nitrogen for protein analysis.

For the in vivo rescue experiments, a 8505C‐ERα‐OE stable cell line was established by lentiviral transduction. ERα‐OE cells (2 × 10^6^ cells in 100 µL of PBS with 100 µL of Matrigel) were injected subcutaneously into the right flank of BALB/c nude mice. When tumors became palpable on Day 7, mice were randomized into five groups (n = 10 per group): (1) Vector (intratumoral injection of empty lentivirus), (2) ERα‐OE (intratumoral injection of empty lentivirus), (3) ERα‐OE + shPADI4 (intratumoral injection of shPADI4‐expressing lentivirus, 1 × 10^8^ TU in 50 µL, twice weekly), (4) ERα‐OE + CXCR2i (SX‐682, MedChemExpress, 100 mg/kg, oral gavage, daily), and (5) ERα‐OE + Anti‐Ly6G (anti‐Ly6G monoclonal antibody, BioXCell clone 1A8, 200 µg/mouse, intraperitoneal injection, twice weekly). Body weights were recorded every 3 days. Mice were euthanized on Day 28, and tumors and lungs were collected for analysis.

For the combination therapy study, the same ERα‐OE subcutaneous model was used. From Day 7 to Day 27, mice received daily oral gavage of Vehicle (0.5% w/v carboxymethylcellulose sodium + 0.1% v/v Tween‐80 in sterile water), GSK484 (50 mg/kg, suspended in Vehicle), SX‐682 (100 mg/kg, suspended in Vehicle), or the combination of both agents at the same respective doses (n = 12 per group). GSK484 was synthesized and validated (>98% purity) by MedChemExpress. Mice were monitored daily, and tumor growth was measured every 3 days. Mice were euthanized on Day 28 (for tumor and lung collection) or followed for survival analysis until Day 80. For survival studies, humane endpoints were defined as tumor volume exceeding 2000 mm^3^, tumor ulceration exceeding 25% of the tumor surface, or body weight loss exceeding 20% of initial weight.

For the subcutaneous tumor model (Figure 2), no animals in any group reached humane endpoints before the Day 30 endpoint. For the in vivo rescue experiments (Figure 5), 3 of 10 mice in the ERα‐OE group were euthanized between Day 24 and Day 26 due to tumor volume exceeding 2000 mm^3^; these animals were counted as events in the survival analysis, and their tumor volume and weight data at the time of euthanasia were carried forward. No animals in the Vector or rescue groups reached humane endpoints before Day 28. For the combination therapy study (Figure 6), no animals in any group reached humane endpoints before the Day 28 endpoint. For the survival study (Figure 6h), all animals euthanized due to reaching humane endpoints were counted as events in the Kaplan‐Meier analysis.

### 2.20 Flow Cytometric Analysis of Tumor‐Infiltrating Immune Cells

Freshly excised tumors were minced into small fragments with scissors and enzymatically digested in RPMI‐1640 containing 1 mg/mL collagenase IV (Sigma‐Aldrich), 0.1 mg/mL DNase I (Sigma‐Aldrich), and 2% FBS at 37°C for 45 min with continuous agitation. The cell suspension was filtered through a 70 µm cell strainer, and red blood cells were lysed with ACK lysis buffer. Cells were washed and resuspended in PBS containing 2 mM EDTA and 2% FBS (FACS buffer). For surface staining, cells were incubated with fluorochrome‐conjugated antibodies against CD45 (APC‐Cy7, clone 30‐F11), CD11b (FITC, clone M1/70), Gr‐1 (APC, clone RB6‐8C5), CD3 (PerCP‐Cy5.5, clone 145‐2C11), CD4 (PE‐Cy7, clone RM4‐5), and CD8 (BV421, clone 53‐6.7) for 30 min at 4°C in the dark. For FoxP3 intracellular staining, cells were fixed and permeabilized using the FoxP3/Transcription Factor Staining Buffer Set (eBioscience) and stained with anti‐FoxP3 antibody (PE, clone FJK‐16s). All antibodies were purchased from BioLegend or BD Biosciences. Flow cytometry was performed on a BD LSRFortessa X‐20 cytometer, and data were analyzed with FlowJo software (v10.8.1). MDSCs were defined as CD45^+^CD11b^+^Gr‐1^+^ cells, CD8^+^ T cells as CD45^+^CD3^+^CD8^+^ cells, and Tregs as CD4^+^FoxP3^+^ cells.

### 2.21 Histopathology and Immunohistochemistry of Tumor and Organ Tissues

Formalin‐fixed, paraffin‐embedded tumor and organ tissues were sectioned at 5 µm thickness and stained with hematoxylin and eosin (H&E) according to standard protocols. For Ki67 IHC, sections were incubated with anti‐Ki67 antibody (CST #9449, 1:400). For cleaved caspase‐3 IHC, anti‐cleaved caspase‐3 (Asp175) antibody (CST #9661, 1:400) was used. For TUNEL staining, the In Situ Cell Death Detection Kit, Fluorescein (Roche) was used according to the manufacturer's protocol. For the visualization of pulmonary metastases, whole lungs were fixed in 10% neutral buffered formalin, embedded in paraffin, and serially sectioned at 200 µm intervals throughout the entire lung. H&E‐stained sections were examined under a light microscope, and the number of metastatic nodules (defined as clusters of >50 tumor cells) per lung was counted. For multi‐organ toxicity assessment, heart, liver, spleen, lung, kidney, and brain from the Combo treatment group were processed identically. Histopathological evaluation was performed in a blinded manner by a board‐certified veterinary pathologist using a semi‐quantitative scoring system (0 = no lesion, 1 = minimal, 2 = mild, 3 = moderate, 4 = severe) for the following parameters: inflammation, necrosis, fibrosis, and vascular changes.

### 2.22 Serum Estradiol and CXCL8 Measurements

Serum estradiol (E2) concentrations were measured using the Estradiol ELISA Kit (Cayman Chemical, item #582251) according to the manufacturer's protocol. The detection limit was 6.6 pg/mL. Serum CXCL8 levels in mice were measured using the Mouse CXCL8/KC Quantikine ELISA Kit (R&D Systems). All ELISA measurements were performed in duplicate, and absorbance was read at 450 nm using a microplate reader with wavelength correction at 540 nm.

### 2.23 Statistical Analysis

All statistical analyses were performed using GraphPad Prism (v9.5) and R (v4.1.2). Data are presented as mean ± standard deviation (SD) or median with interquartile range (IQR) for non‐normally distributed variables. Normal distribution of data was assessed using the Shapiro‐Wilk test. For comparisons between two groups, unpaired two‐tailed Student's t‐test was used for normally distributed data, and Mann‐Whitney U test was used for non‐normally distributed data. For comparisons among three or more groups, one‐way analysis of variance (ANOVA) followed by Tukey's post‐hoc test was used for normally distributed data, and Kruskal‐Wallis test followed by Dunn's post‐hoc test was used for non‐normally distributed data. For repeated measurements (e.g., tumor growth curves), two‐way repeated measures ANOVA was used with Greenhouse‐Geisser correction when sphericity was violated. Correlations were assessed using Pearson's r (for normally distributed variables) or Spearman's rank correlation coefficient (for non‐normally distributed variables).

Overall survival was defined as the time from diagnosis to death from any cause or last follow‐up. For the generation of Kaplan‐Meier curves, the optimal cutoff value for PADI4 IHC H‐score to stratify patients into PADI4‐High and PADI4‐Low groups was determined using the median H‐score (cutoff = 120) of the 78‐patient ATC cohort. Survival curves were estimated using the Kaplan‐Meier method and compared using the log‐rank test. Median survival times with 95% confidence intervals were calculated for each treatment group. For the multivariate Cox proportional hazards regression model, age was treated as a continuous variable, PADI4 H‐score was treated as a categorical variable (High vs. Low based on the median cutoff), sex was treated as a categorical variable, and TNM Stage was treated as a categorical variable (Stage IV vs. Stages I–III). Variables with *p <* 0.10 in the univariate analysis were included in the multivariate model to identify independent prognostic factors.

Receiver operating characteristic (ROC) curves were generated to evaluate the diagnostic performance of PADI4 IHC for discriminating ATC from PTC, and the area under the curve (AUC) was calculated with 95% CI. The combination index (CI) for drug synergy was calculated using the Chou‐Talalay method with CompuSyn software (v1.0), where CI < 1 indicates synergy, CI = 1 indicates additivity, and CI > 1 indicates antagonism. A two‐sided *p*‐value < 0.05 was considered statistically significant. All experiments were performed independently at least three times unless otherwise specified in the figure legends. The sample sizes for each experiment are indicated in the respective figure legends or method descriptions.

## 3 Results

### 3.1 PADI4 Is Overexpressed in ATC in a Ligand‐Independent Manner and Defines an Immunosuppressive Microenvironment With Poor Prognosis

To establish the clinical relevance of the PADI4/ERα axis in anaplastic thyroid carcinoma (ATC), we assembled a multi‐cohort transcriptomic and tissue microarray dataset encompassing normal thyroid (n = 59), papillary thyroid carcinoma (PTC, n = 365), poorly differentiated thyroid carcinoma (PDTC, n = 28), and ATC (n = 65). PADI4 mRNA expression exhibited a stepwise increase along the dedifferentiation spectrum, with ATC showing the highest levels (median log_2_ TPM = 6.8) compared with PDTC (4.8), PTC (3.2) and normal tissue (1.1) (*p <* 0.001, one‐way ANOVA with Tukey post‐hoc test; Figure 1a). In striking contrast, ESR1 mRNA encoding estrogen receptor alpha (ERα) remained unchanged across all groups (median approximately 5.0 for all; *p =* 0.32; Figure 1b), suggesting that ERα activity in ATC is regulated at the post‐translational rather than transcriptional level. Immunoblotting of fresh‐frozen clinical specimens confirmed this discordance: p‐ERα (S118) and PADI4 protein levels were elevated approximately 8‐fold and 6.5‐fold, respectively, in ATC compared with normal and PTC tissues, whereas total ERα protein abundance was comparable across groups, and GAPDH loading controls were uniform (Figure 1c). Immunohistochemistry (IHC) on an independent tissue microarray corroborated these findings, with PADI4 H‐scores of 185.6 ± 52.3 in ATC (n = 35) versus 45.2 ± 22.1 in PTC (n = 40) and 2.1 ± 1.5 in normal thyroid (n = 20) (*p <* 0.001; Figure 1d,e). Notably, a significant positive correlation was observed between ERα and PADI4 IHC scores in ATC specimens (Pearson r = 0.68, *p <* 0.001; Figure 1f).

**FIGURE 1:**
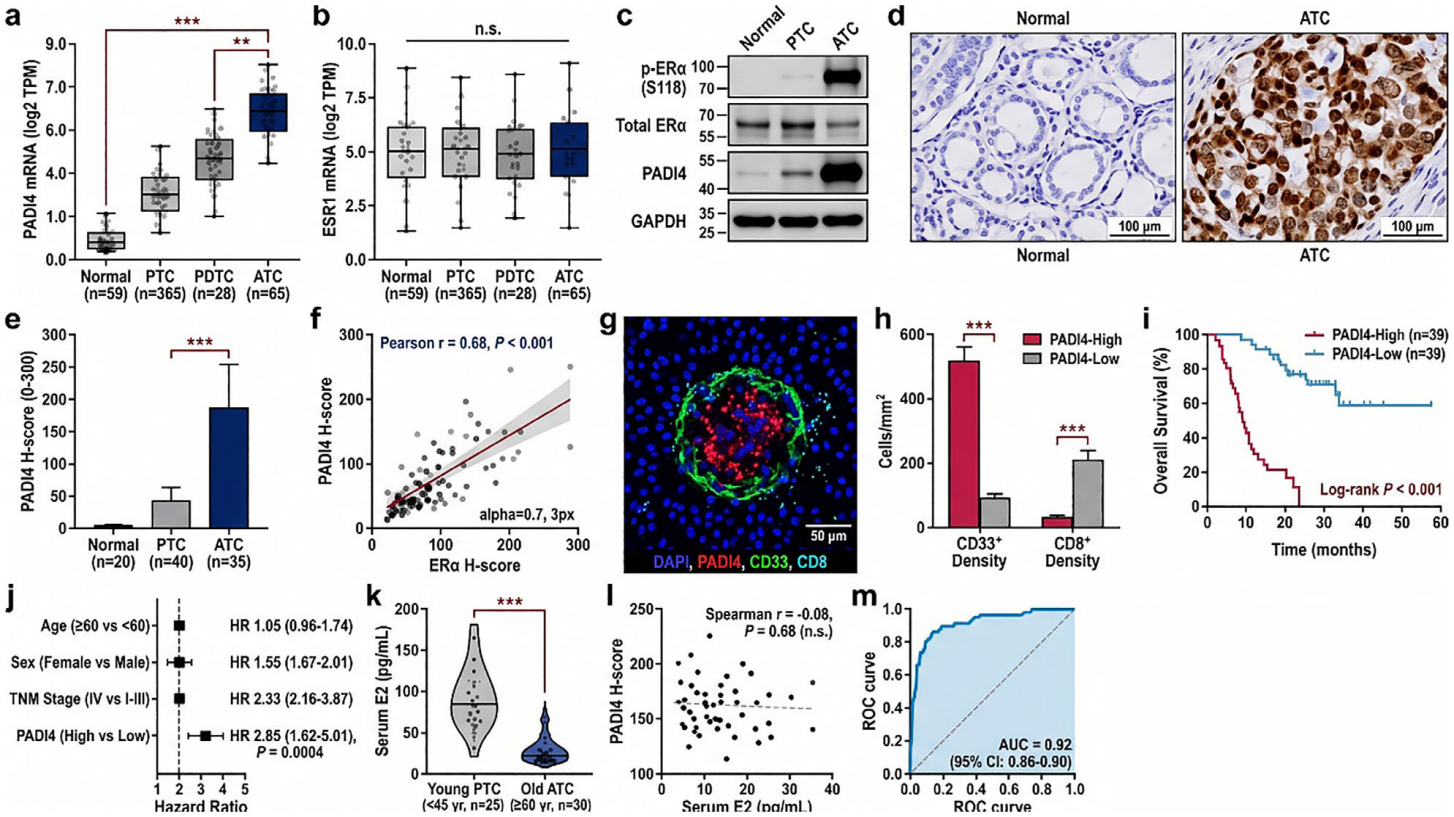
PADI4 is overexpressed in ATC via ligand‐independent ERα activation and correlates with an immunosuppressive microenvironment and poor prognosis. (a) Box plot of PADI4 mRNA expression (log_2_ TPM) across normal thyroid (n = 59), PTC (n = 365), PDTC (n = 28), and ATC (n = 65). ATC shows the highest expression. ***p < 0.001 vs. all other groups; **p = 0.0038 for ATC vs. PDTC (one‐way ANOVA, Tukey post‐hoc). (b) Box plot of ESR1 (ERα) mRNA showing no significant difference among groups (n.s.,, not significant; *p =* 0.32). (c) Representative western blot of clinical tissue lysates for p‐ERα (S118), total ERα, PADI4, and GAPDH. ATC lanes show markedly elevated p‐ERα and PADI4 bands (∼8‐fold and ∼6.5‐fold vs. Normal, respectively). p‐ERα and total ERα migrate at approximately 66 kDa, PADI4 at approximately 74 kDa, and GAPDH at approximately 37 kDa. Molecular weight markers (kDa) indicated at left. (d) Representative PADI4 IHC images of normal thyroid and ATC tissues. Scale bar, 100 µm. (e) Quantification of PADI4 H‐score in normal (n = 20), PTC (n = 40), and ATC (n = 35) tissues. ATC: 185.6 ± 52.3. ***p < 0.001. (f) Correlation between ERα and PADI4 IHC H‐scores in ATC specimens (n = 70 regions of interest). Pearson r = 0.68, *p <* 0.001. (g) Representative multiplex immunofluorescence image of ATC tissue showing DAPI (blue nuclei), PADI4 (red puncta), CD33 (green, MDSCs), and CD8 (cyan, T cells). Note the dense CD33^+^ ring surrounding PADI4‐high areas and CD8^+^ exclusion. Scale bar, 50 µm. (h) Quantification of CD33^+^ and CD8^+^ cell densities in PADI4‐high vs. PADI4‐low regions. ***p < 0.001. (i) Kaplan‐Meier overall survival curves for ATC patients stratified by median PADI4 IHC H‐score (PADI4‐High, n = 39; PADI4‐Low, n = 39). Log‐rank *p <* 0.001. Risk table below. (j) Multivariate Cox regression forest plot. PADI4: HR = 2.85 (95% CI 1.62–5.01), *p =* 0.0004. (k) Serum E2 levels in young PTC (<45 y, n = 25) vs. elderly ATC (≥60 y, n = 30) patients. ***p < 0.001 (Mann‐Whitney U test). (l) Correlation between serum E2 and tumor PADI4 H‐score in elderly ATC patients. Spearman r = −0.08, *p =* 0.68 (n.s.). (m) ROC curve for PADI4 IHC discriminating ATC from PTC. AUC = 0.92 (95% CI 0.86–0.98). Data are mean ± SD or n (%). P‐values from unpaired t‐test or χ^2^ test. For all panels, *p < 0.05, **p < 0.01, ***p < 0.001; n.s., not significant.

Multiplex immunofluorescence (mIF) revealed that PADI4‐high regions within ATC tissues were spatially surrounded by a dense annulus of CD33^+^ myeloid‐derived suppressor cells (MDSCs) while CD8^+^ cytotoxic T cells were largely excluded, residing predominantly in the PADI4‐low periphery (Figure 1g). Quantitative image analysis confirmed that PADI4‐high zones harbored markedly elevated CD33^+^ cell density (520 ± 88 vs. 95 ± 32 cells/mm^2^; *p <* 0.001) and profoundly depleted CD8^+^ density (28 ± 12 vs. 210 ± 45 cells/mm^2^; *p <* 0.001) compared with PADI4‐low zones (Figure 1h). These spatial patterns are indicative of an immune‐excluded, immunosuppressive tumor microenvironment. Kaplan‐Meier analysis of 78 ATC patients stratified by median PADI4 IHC H‐score demonstrated that the PADI4‐high group had a dramatically shorter median overall survival (7.8 months vs. 32.5 months; log‐rank *p <* 0.001; Figure 1i). Multivariate Cox regression confirmed PADI4 as an independent prognostic factor (HR = 2.85, 95% CI 1.62–5.01, *p =* 0.0004) after adjusting for age, sex, and TNM stage (Figure 1j).

Given that ATC predominantly affects elderly postmenopausal women, we measured serum estradiol (E2) levels and found that elderly ATC patients (≥60 years) had profoundly low circulating E2 (median 12 pg/mL) compared with young PTC patients (<45 years, median 88 pg/mL; *p <* 0.001; Figure 1k). Importantly, within the elderly ATC cohort, serum E2 concentrations showed no correlation with tumor PADI4 H‐score (Spearman r = −0.08, *p =* 0.68; Figure 1l). These data collectively argue that PADI4 upregulation in ATC occurs through a ligand‐independent mechanism, consistent with the preserved p‐ERα (S118) levels despite negligible circulating estrogen. The diagnostic utility of PADI4 IHC for distinguishing ATC from PTC was excellent (AUC = 0.92, 95% CI 0.86–0.98; Figure 1m). Finally, baseline clinicopathological characteristics revealed that PADI4‐high tumors were significantly larger, more frequently exhibited extrathyroidal extension, lymph node metastasis, and distant metastasis, and were predominantly Stage IV (all *p <* 0.001), while age and sex distributions were comparable between PADI4‐high and PADI4‐low groups (Figure S1). Collectively, these findings establish PADI4 as a clinically relevant biomarker that defines an immunosuppressive niche and portends dismal prognosis in ATC, with its expression driven by ligand‐independent ERα activation rather than circulating estrogen.

### 3.2 PADI4 Maintains the Malignant Phenotype of ATC and Orchestrates an Immunosuppressive Microenvironment

To functionally dissect the role of PADI4 in ATC, we first profiled its mRNA expression across a panel of four authenticated ATC cell lines (8505C, CAL‐62, BHT‐101, and KTC‐1). The monocytic leukemia cell line THP‐1 was included as a hematopoietic reference, given the known expression of PADI4 in myeloid lineage cells and the focus of this study on ATC‐MDSC crosstalk. Among the ATC lines, 8505C and CAL‐62 exhibited the highest PADI4 expression and were selected for further study (Figure 2a). Two independent shRNAs targeting PADI4 (sh‐PADI4 #1 and #2) achieved near‐complete silencing at the protein level relative to the non‐targeting control (sh‐NC), with GAPDH confirming equal loading (Figure 2b). CCK‐8 proliferation assays revealed that PADI4 depletion profoundly suppressed cell growth, with sh‐NC cells reaching an OD of ∼1.8 at Day 5 compared with only ∼0.55 for sh‐PADI4 cells (*p <* 0.001 from Day 3 onward; Figure 2c). This proliferative defect was corroborated by EdU incorporation assays, which showed a reduction in the fraction of EdU‐positive cells from 58.3 ± 6.1% in sh‐NC to 14.7 ± 3.8% in sh‐PADI4 cultures (*p <* 0.001; Figure 2d), and by colony formation assays, in which sh‐PADI4 cells formed only sparse, minute colonies compared with the abundant, large colonies of sh‐NC cells (Figure 2e). Transwell migration and Matrigel invasion assays further demonstrated that PADI4 knockdown significantly impaired the migratory (∼85% reduction) and invasive (∼86% reduction) capacity of ATC cells (Figures 2f,g). Consistently, wound healing was markedly delayed in sh‐PADI4 monolayers (18.3 ± 5.6% closure at 24 h vs. 82.5 ± 7.3% for sh‐NC; *p <* 0.001; Figure 2h).

**FIGURE 2:**
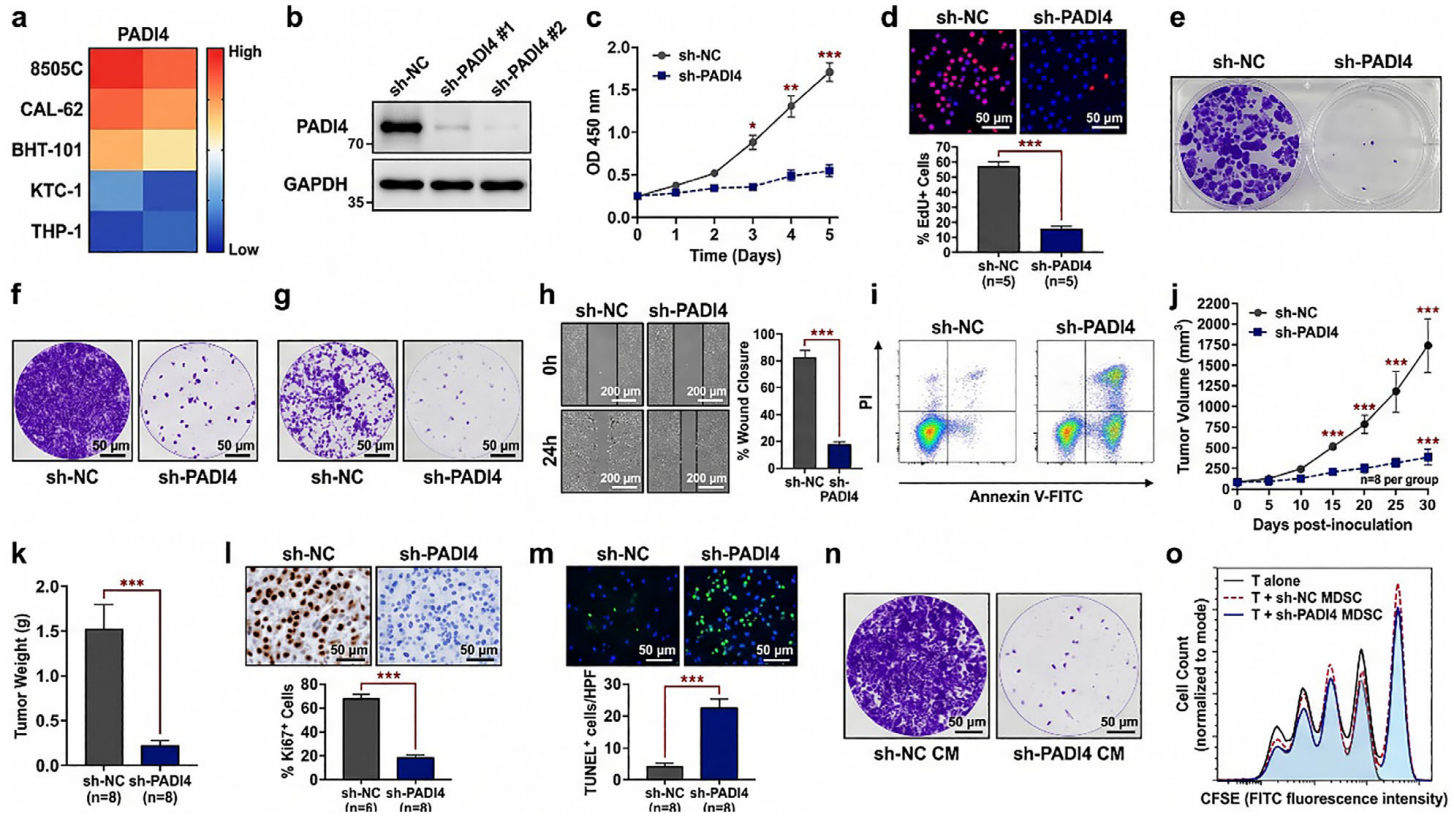
PADI4 is required for the malignant phenotype of ATC cells and promotes an immunosuppressive microenvironment via MDSC‐mediated T‐cell suppression. (a) Heatmap of PADI4 mRNA expression in four ATC cell lines (8505C, CAL‐62, BHT‐101, KTC‐1) and the monocytic leukemia cell line THP‐1, included as a hematopoietic reference. Data are row Z‐score normalized ΔCt values (normalized to GAPDH). Color scale from deep blue (low expression) through white to bright red (high expression). (b) Western blot validation of PADI4 knockdown efficiency in 8505C cells using two independent shRNAs. GAPDH serves as loading control. Molecular weight markers (kDa) indicated at left. (c) CCK‐8 proliferation curves of sh‐NC and sh‐PADI4 8505C cells over 5 days. *p < 0.05, **p < 0.01, ***p < 0.001 vs. sh‐NC at each time point. (d) Representative EdU immunofluorescence images (top) and quantification of EdU^+^ cells (bottom). Scale bar, 50 µm. n = 5 fields per group. ***p < 0.001. (e) Representative images of colony formation assay. Crystal violet staining at 14 days. (f,g) Representative Transwell migration (f) and Matrigel invasion (g) images of sh‐NC and sh‐PADI4 cells. Scale bar, 50 µm. (h) Wound healing assay showing representative phase‐contrast images at 0 and 24 h (left) and quantification of wound closure (right). Scale bar, 200 µm. ***p < 0.001. (i) Flow cytometric analysis of apoptosis by Annexin V‐FITC/PI dual staining. Quadrants: Q3, live; Q4, early apoptotic; Q2, late apoptotic. The total apoptotic fraction is annotated. (j) Subcutaneous tumor growth curves in BALB/c nude mice. n = 8 per group. ***p < 0.001 from Day 15 onward. (k) Endpoint tumor weights at Day 30. ***p < 0.001. (l) Representative Ki67 IHC images (top) and quantification (bottom) of excised tumors. Scale bar, 50 µm. n = 8. ***p < 0.001. (m) Representative TUNEL immunofluorescence images (top) and quantification (bottom). Scale bar, 50 µm. n = 8. ***p < 0.001. (n) Transwell MDSC chemotaxis assay using conditioned medium (CM) from sh‐NC or sh‐PADI4 ATC cells. CM: serum‐free, 48 h. MDSC: mouse bone marrow‐derived CD11b^+^Gr‐1^+^ cells. Scale bar, 50 µm. (o) Overlaid CFSE flow cytometry histograms of CD8^+^ T cells cultured alone (T only, black solid), with sh‐NC‐educated MDSCs (T + sh‐NC MDSC, red dashed), or with sh‐PADI4‐educated MDSCs (T + sh‐PADI4 MDSC, blue solid). All data are presented as mean ± SD. *p < 0.05, **p < 0.01, ***p < 0.001; n.s., not significant.

Flow cytometric analysis using Annexin V‐FITC/PI staining uncovered a significant increase in apoptosis following PADI4 depletion, with the total apoptotic fraction rising from approximately 5.3% in sh‐NC cells to 20.8% in sh‐PADI4 cells (*p <* 0.001; Figure 2i). We next extended these observations to an in vivo xenograft model. Subcutaneous implantation of sh‐NC 8505C cells into BALB/c nude mice produced rapidly growing tumors that reached a mean volume of ∼1750 mm^3^ and a weight of 1.52 ± 0.18 g by Day 30, whereas sh‐PADI4 tumors were dramatically smaller, reaching only ∼280 mm^3^ and weighing 0.22 ± 0.07 g (*p <* 0.001; Figure 2j,k). Immunohistochemical analysis of excised tumors confirmed that the proliferative index was significantly lower in sh‐PADI4 tumors (Ki67 positivity: 18.2 ± 5.1% vs. 68.5 ± 8.2% in sh‐NC; *p <* 0.001; Figure 2l), while TUNEL staining revealed a corresponding increase in apoptosis (22.5 ± 4.3 vs. 3.2 ± 1.1 apoptotic cells/HPF; *p <* 0.001; Figure 2m). Crucially, conditioned medium from sh‐PADI4 ATC cells exhibited a profoundly diminished capacity to support MDSC chemotaxis in Transwell assays compared with sh‐NC conditioned medium (*p <* 0.001; Figure 2n). This defect in MDSC recruitment translated into a functional consequence: in CFSE‐based T‐cell proliferation assays, MDSCs educated by sh‐NC conditioned medium almost completely arrested CD8^+^ T‐cell proliferation, whereas MDSCs educated by sh‐PADI4 conditioned medium failed to do so, allowing T cells to undergo multiple rounds of division comparable to T cells cultured alone (Figure 2o). Quantitative analysis of the Division Index from three independent experiments confirmed a significant restoration of T‐cell proliferative capacity in the sh‐PADI4 MDSC co‐culture condition (Division Index: 1.89 ± 0.28 vs. 0.18 ± 0.06 for sh‐NC MDSC; *p <* 0.001; Table S1). Collectively, these findings establish that PADI4 is essential for sustaining the proliferative, migratory, invasive, and pro‐metastatic properties of ATC cells and, equally importantly, that PADI4 endows ATC cells with the capacity to instruct MDSCs to acquire an immunosuppressive phenotype that cripples cytotoxic T‐cell responses.

### 3.3 PADI4 Promotes CXCL8 Transcription via Histone Citrullination to Recruit MDSCs, With Compensatory CXCL1 Upregulation Upon Pharmacological Inhibition

To identify the downstream effectors through which PADI4 shapes the immunosuppressive microenvironment, we performed RNA‐sequencing of 8505C cells transduced with sh‑NC or sh‑PADI4. Among the 1250 differentially expressed genes, *CXCL8* emerged as the most profoundly downregulated chemokine (log_2_ fold change = –3.2, adjusted *p =* 5.2 × 10^−^^13^), with several other CXC chemokines also markedly reduced (Figure 3a). Global transcriptomic heatmap analysis further revealed that PADI4 depletion broadly suppressed chemokine and cytokine genes (e.g., *CXCL8, CXCL1, CXCL2, CXCL5, CCL20, CSF2, IL6, MMP9*) while concurrently upregulating apoptosis‐ and stress‐response genes (e.g., *CDKN1A, GADD45B, DDIT3, BBC3, FAS*), identifying PADI4 as a dual‐function transcriptional regulator that simultaneously promotes an immunosuppressive secretome and suppresses apoptotic programs in ATC cells (Figure S2). Quantitative PCR confirmed the massive suppression of *CXCL8* mRNA (≈45‑fold decrease; *p <* 0.001) and, notably, revealed a compensatory upregulation of *CXCL1* (≈2.5‑fold increase; *p =* 0.003) (Figure 3b). At the protein level, *CXCL8* secretion into conditioned medium dropped by approximately 88% in sh‑PADI4 cells (820 ± 65 vs. 95 ± 22 pg/mL/10^6^ cells; *p <* 0.001) (Figure 3c). Functionally, conditioned medium from sh‑PADI4 cells lost its ability to chemoattract bone marrow‑derived MDSCs in Transwell assays, and this defect was fully rescued by supplementation with recombinant human *CXCL8* (50 ng/mL), unequivocally establishing *CXCL8* as the principal PADI4‑dependent chemoattractant for MDSCs (Figure 3d,e). Blockade of the *CXCL8* receptor *CXCR2* with a neutralizing antibody completely abrogated the rescued chemotaxis, confirming that the *CXCL8‑CXCR2* axis mediates MDSC recruitment (Figure 3f).

**FIGURE 3:**
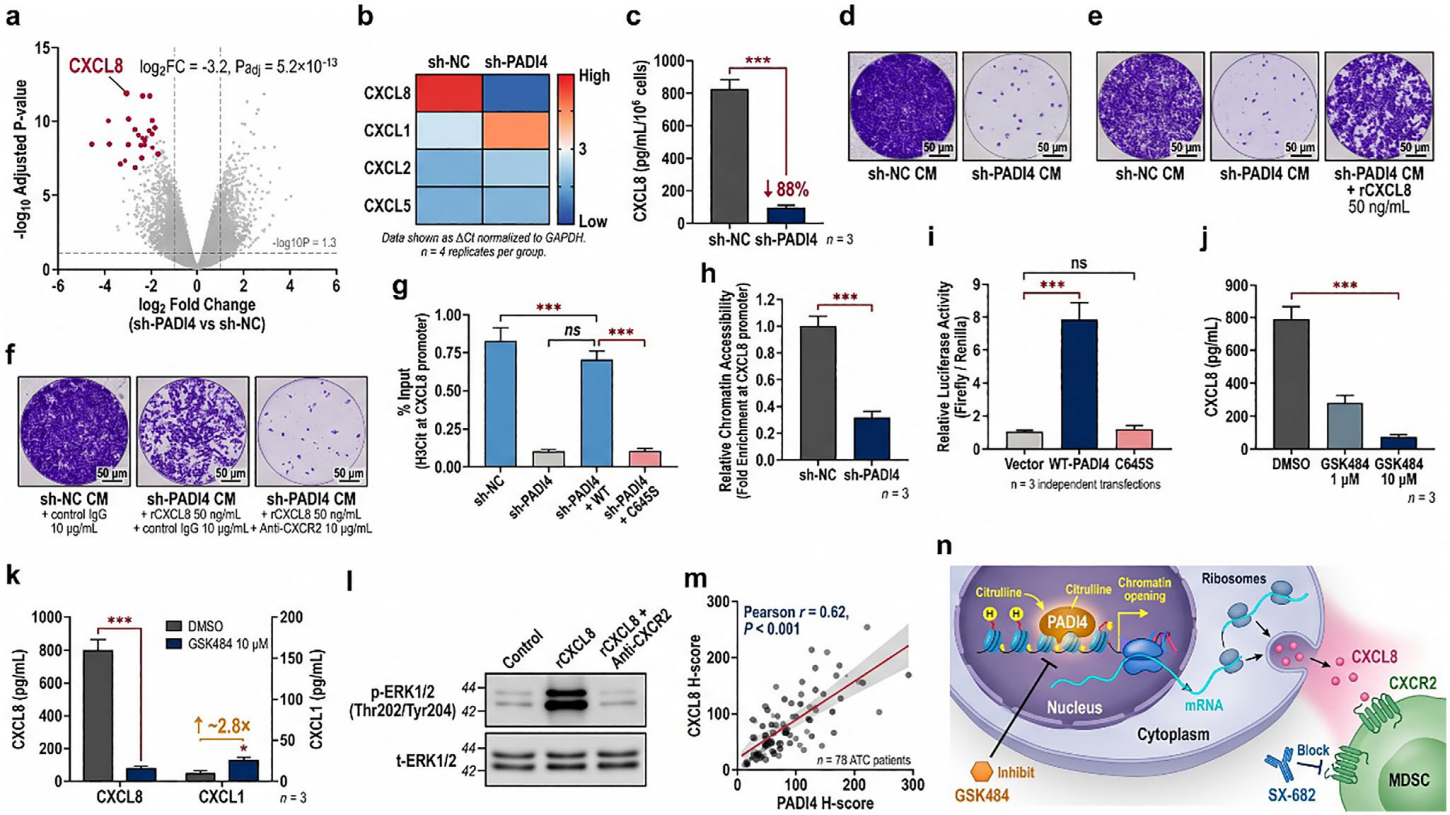
PADI4 drives CXCL8 transcription through citrullination of histone H3 at the CXCL8 promoter to recruit MDSCs via CXCR2, with compensatory CXCL1 upregulation upon PADI4 inhibition. (a) RNA‑seq volcano plot of sh‑PADI4 vs. sh‑NC 8505C cells. Significantly downregulated genes are shown in red. CXCL8 is highlighted. Dashed lines indicate thresholds of |log_2_FC| = 1.0 and adjusted *p =* 0.05. (b) qPCR heatmap of selected chemokines. Data are row Z‐score normalized ΔCt values (normalized to GAPDH). n = 4 replicates. (c) ELISA quantification of CXCL8 secretion. ***p < 0.001; n = 3. (d) Transwell MDSC chemotaxis using conditioned medium (CM) from sh‑NC or sh‑PADI4 cells. Scale bar, 50 µm. (e) Chemotaxis rescue assay with recombinant CXCL8 (rCXCL8, 50 ng/mL). Scale bar, 50 µm. (f) CXCR2 blocking assay. Where indicated, MDSCs were pre‑treated with anti‑CXCR2 (10 µg/mL) or control IgG. Scale bar, 50 µm. (g) ChIP‑qPCR for H3Cit at the CXCL8 promoter in four groups: sh‑NC, sh‑PADI4, sh‑PADI4 rescued with wild‑type PADI4 (WT), and with the catalytically inactive C645S mutant. ***p < 0.001; ns, not significant; n = 3. C645 is the catalytic residue of human PADI4. (h) ATAC‑qPCR at the CXCL8 locus. ***p < 0.001; n = 3. (i) Dual‑luciferase reporter assay using the CXCL8 promoter (–500/+50 bp). ***p < 0.001; ns, not significant; n = 3. (j) Dose‑dependent inhibition of CXCL8 secretion by GSK484. ***p < 0.001 for linear trend; n = 3. (k) Compensatory increase in CXCL1 secretion upon GSK484 treatment (10 µM). *p < 0.05, ***p < 0.001; n = 3. (l) Western blot for p‑ERK1/2 and total ERK1/2 in MDSCs stimulated with rCXCL8 ± anti‑CXCR2. Molecular weight markers indicated. (m) Correlation of PADI4 and CXCL8 IHC scores in 78 ATC specimens. Pearson r = 0.62, *p <* 0.001. (n) Three‑dimensional mechanistic schematic summarizing the PADI4–H3Cit–CXCL8–CXCR2–MDSC pathway and the compensatory CXCL1 axis, with GSK484 and SX‑682 intervention points. Data are mean ± SD.

Mechanistically, chromatin immunoprecipitation coupled with quantitative PCR (ChIP‑qPCR) for citrullinated histone H3 (H3Cit) revealed that PADI4 directly citrullinates histones at the *CXCL8* promoter, with H3Cit enrichment at 0.82 ± 0.09% of input in sh‑NC cells being reduced to 0.09 ± 0.02% upon PADI4 knockdown (*p <* 0.001). Crucially, re‑expression of wild‑type PADI4 in knockdown cells restored H3Cit enrichment to 0.71 ± 0.08% (not significantly different from sh‑NC), whereas the catalytically dead C645S mutant failed to do so (0.10 ± 0.03%; *p <* 0.001 vs. WT rescue), demonstrating that the citrullination activity of PADI4 is absolutely required for this epigenetic modification (Figure 3g). Consistent with the loss of H3Cit, ATAC‑qPCR showed a 69% reduction in chromatin accessibility at the *CXCL8* locus in sh‑PADI4 cells relative to sh‑NC controls (*p <* 0.001) (Figure 3h). In dual‑luciferase reporter assays, wild‑type PADI4 transactivated the *CXCL8* promoter approximately 7.8‑fold, whereas the C645S mutant was completely inert (Figure 3i). Pharmacological inhibition of PADI4 with GSK484 phenocopied the genetic knockdown in a dose‑dependent manner, reducing *CXCL8* secretion from 795 ± 58 pg/mL (DMSO) to 72 ± 18 pg/mL at 10 µM (*p <* 0.001 for trend) (Figure 3j), and concurrently triggering a compensatory rise in CXCL1 secretion from 45 ± 12 to 128 ± 28 pg/mL (*p <* 0.05; Figure 3k). We note that the compensatory *CXCL1* upregulation is inferred from combined genetic (mRNA) and pharmacological (protein) evidence; direct CXCL1 protein data from the sh‐PADI4 genetic knockdown condition were not obtained in this study, representing a minor gap in the protein‐level correspondence that should be addressed in future work. Downstream of CXCR2, recombinant *CXCL8* potently activated ERK1/2 phosphorylation in MDSCs, an effect entirely blocked by anti‑CXCR2 (Figure 3l). Finally, the clinical relevance of this axis was underscored by a significant positive correlation between PADI4 and *CXCL8* IHC scores in 78 ATC patient specimens (Pearson r = 0.62, *p <* 0.001) (Figure 3m). An integrated three‑dimensional mechanistic model summarizing the PADI4–H3Cit–CXCL8–CXCR2–MDSC signaling cascade and the compensatory CXCL1 axis is presented in Figure 3n.

### 3.4 Ligand‐Independent Phosphorylation of ERα at S118 by the MAPK Pathway Drives PADI4 Transcription in ATC

Given that circulating estradiol is negligible in elderly ATC patients (Figure 1k), we hypothesized that ERα is activated through ligand‐independent mechanisms. Stimulation of serum‐starved 8505C cells with EGF (50 ng/mL) induced rapid phosphorylation of ERα at S118, which peaked at 15–30 min, while PADI4 protein accumulated progressively and reached its maximum at 120 min—a temporal sequence consistent with p‐ERα acting upstream of PADI4 (Figure 4a). Pretreatment with the MEK inhibitor U0126 (10 µM) completely abrogated EGF‐induced p‐ERα and PADI4 upregulation, restoring both to basal levels (Figure 4b), directly implicating the MAPK/ERK cascade. Concordantly, transcriptomic pathway enrichment analysis of ATC tissues identified the MAPK signaling pathway as the most significantly activated upstream cascade (Figure 4c). Chromatin immunoprecipitation coupled with quantitative PCR demonstrated that endogenous ERα physically occupies the PADI4 promoter, with enrichment of 3.52 ± 0.41% of input compared with 0.05 ± 0.02% for IgG control (*p <* 0.001; Figure 4d). Immunofluorescence co‐localization analysis provided cytological evidence supporting this finding: upon EGF stimulation, ERα and PADI4 exhibited prominent nuclear co‐localization in ATC cells, with distinct yellow/orange foci visible in merged images (Figure S3). Quantitative analysis of confocal images from three independent experiments confirmed significant nuclear co‐localization (Pearson's correlation coefficient = 0.62 ± 0.11; Manders' overlap coefficient = 0.71 ± 0.09; Table S2). Dual‐luciferase reporter assays further showed that ERα overexpression transactivated the wild‐type PADI4 promoter by approximately 6.5‐fold, whereas mutation of the core estrogen response element (ERE) motif completely abolished this activation (Figure 4e). Knockdown of ERα by siRNA reduced PADI4 mRNA to 25% of control levels (*p <* 0.001) with a concomitant decline in PADI4 protein (Figure 4f), and pharmacological degradation of ERα with fulvestrant (1 µM, 48 h) phenocopied this effect, causing a near‐complete loss of PADI4 protein (Figure 4g). A cycloheximide chase experiment revealed that the half‐life of PADI4 protein was unaffected by ERα overexpression (approximately 4.5 h in both Vector and ERα‐OE groups; *p =* 0.51), confirming that ERα regulates PADI4 at the transcriptional rather than post‐translational level (Figure 4h). Immunofluorescence microscopy captured the mechanistic basis of this regulation: under serum‐free conditions, ERα was diffusely distributed in the cytoplasm, but EGF stimulation for 30 min drove its complete nuclear translocation (Figure 4i). Clinically, p‐ERα (S118) and PADI4 IHC scores were positively correlated in ATC specimens (Pearson r = 0.55, *p <* 0.001; Figure 4j), and pan‐cancer TCGA data revealed a consistent, albeit modest, positive correlation between ESR1 and PADI4 mRNA levels (Spearman r = 0.31, *p =* 2.1 × 10^−^^8^; Figure 4k). A three‐dimensional schematic summarizing the EGF/MAPK/p‐ERα/PADI4 transcriptional axis is provided in Figure 4l.

**FIGURE 4:**
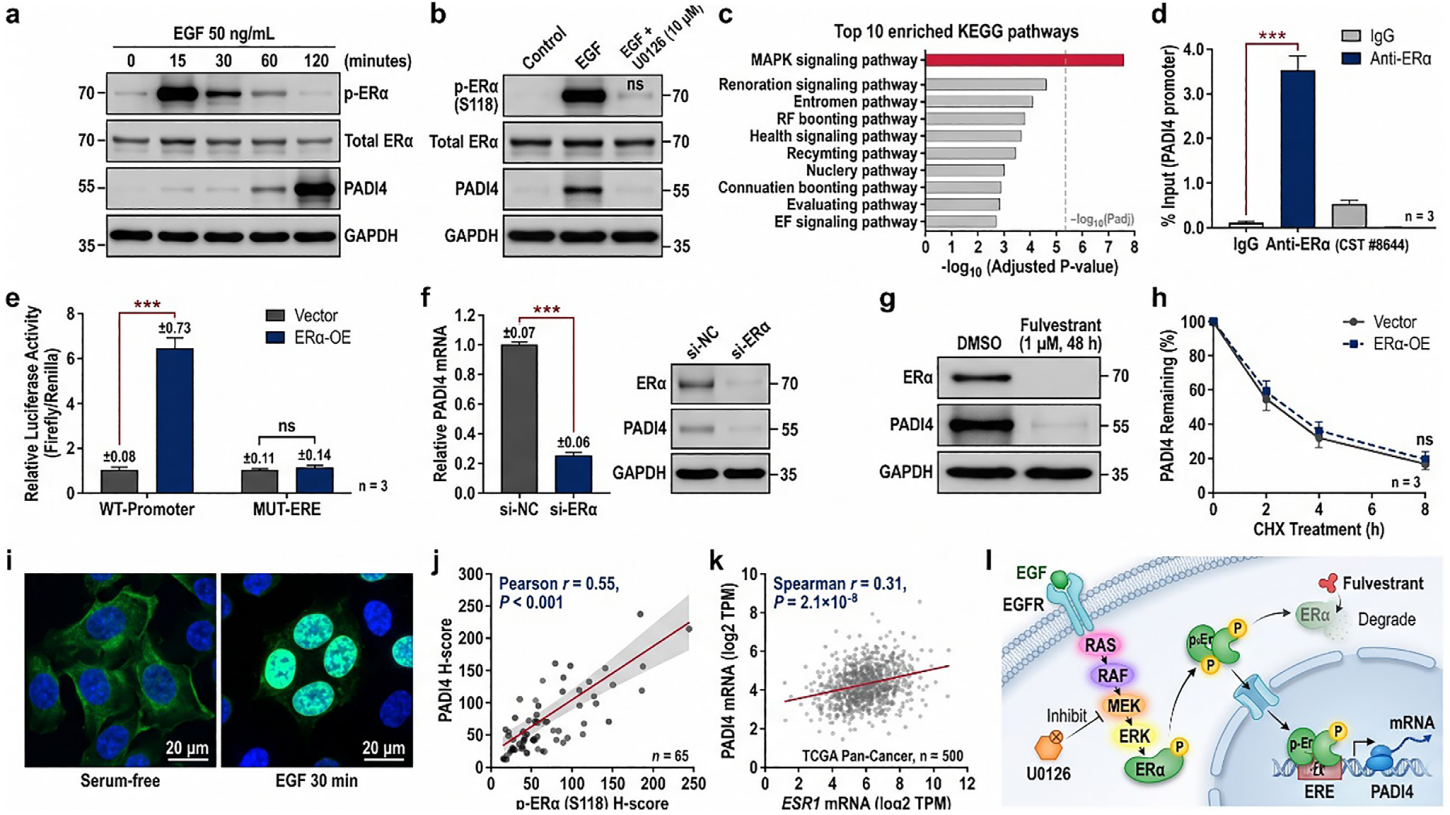
Ligand‐independent ERα S118 phosphorylation by EGF/MAPK signaling directly activates PADI4 transcription. (a) Western blot time course of 8505C cells stimulated with EGF (50 ng/mL). p‐ERα (S118) peaks at 15–30 min; PADI4 peaks at 120 min. GAPDH, loading control. Molecular weight markers indicated. (b) MEK inhibitor U0126 (10 µM) pretreatment abolishes EGF‐induced p‐ERα and PADI4 upregulation. ns, not significant vs. Control. (c) KEGG pathway enrichment analysis of ATC transcriptomes. The MAPK signaling pathway shows the highest enrichment. Dashed line, adjusted *p =* 0.05. (d) ChIP‐qPCR showing ERα binding to the PADI4 promoter. *** *p <* 0.001; n = 3. The antibody clone (CST #8644) is indicated. (e) Dual‐luciferase reporter assays with wild‐type (WT) or estrogen response element‐mutated (MUT‐ERE) PADI4 promoter. *** *p <* 0.001; ns, not significant; n = 3. (f) PADI4 mRNA (left) and protein (right) levels after siRNA‐mediated ERα knockdown. *** *p <* 0.001; n = 3. (g) Western blot showing PADI4 protein after ERα degradation by fulvestrant (1 µM, 48 h). (h) Cycloheximide chase assay: PADI4 protein half‐life is comparable in Vector and ERα‐OE cells. n = 3. (i) Confocal immunofluorescence showing EGF‐induced nuclear translocation of ERα. Scale bar, 20 µm. (j) Correlation between p‐ERα (S118) and PADI4 IHC scores in ATC specimens (n = 65). Pearson r = 0.55, *p <* 0.001. (k) Pan‐cancer TCGA correlation between ESR1 and PADI4 mRNA. Spearman r = 0.31, *p =* 2.1 × 10^−^^8^. (l) Three‐dimensional mechanistic schematic of the EGF/MAPK/p‐ERα/PADI4 transcriptional axis and pharmacological intervention points. Data are mean ± SD.

### 3.5 In Vivo Rescue of the ERα–PADI4–CXCL8–MDSC Signaling Axis Restores Immunosurveillance and Suppresses ATC Progression

To determine whether the ERα–PADI4–CXCL8–MDSC signaling axis is functionally required for ATC progression in vivo, we established an ERα‐overexpressing orthotopic model (8505C‐ERα‐OE) and performed node‐specific rescue experiments. A schematic of the experimental design is shown in Figure 5a, with ten mice per group. ERα‐OE tumors grew aggressively, reaching a mean volume of approximately 1850 mm^3^ and a weight of 1.62 ± 0.22 g by day 28. In stark contrast, silencing PADI4 with intratumoral lentiviral shRNA (+shPADI4), blocking the CXCL8–CXCR2 chemokine axis with the small‐molecule inhibitor SX‐682 (+CXCR2i), or depleting MDSCs with an anti‐Ly6G antibody (+Anti‐Ly6G) each profoundly suppressed tumor growth, with endpoint volumes ranging from 150 to 300 mm^3^ and tumor weights between 0.15 and 0.22 g (all P < 0.001 versus ERα‐OE; Figure 5b–d). These therapeutic effects were accompanied by a marked reduction in the Ki67 proliferation index—from 72.5% in ERα‐OE tumors to 15%–25% across all rescue groups—and a corresponding increase in cleaved caspase‐3‐positive apoptotic cells (from 3.5% to 22%–35%) (Figure 5e,f).

**FIGURE 5:**
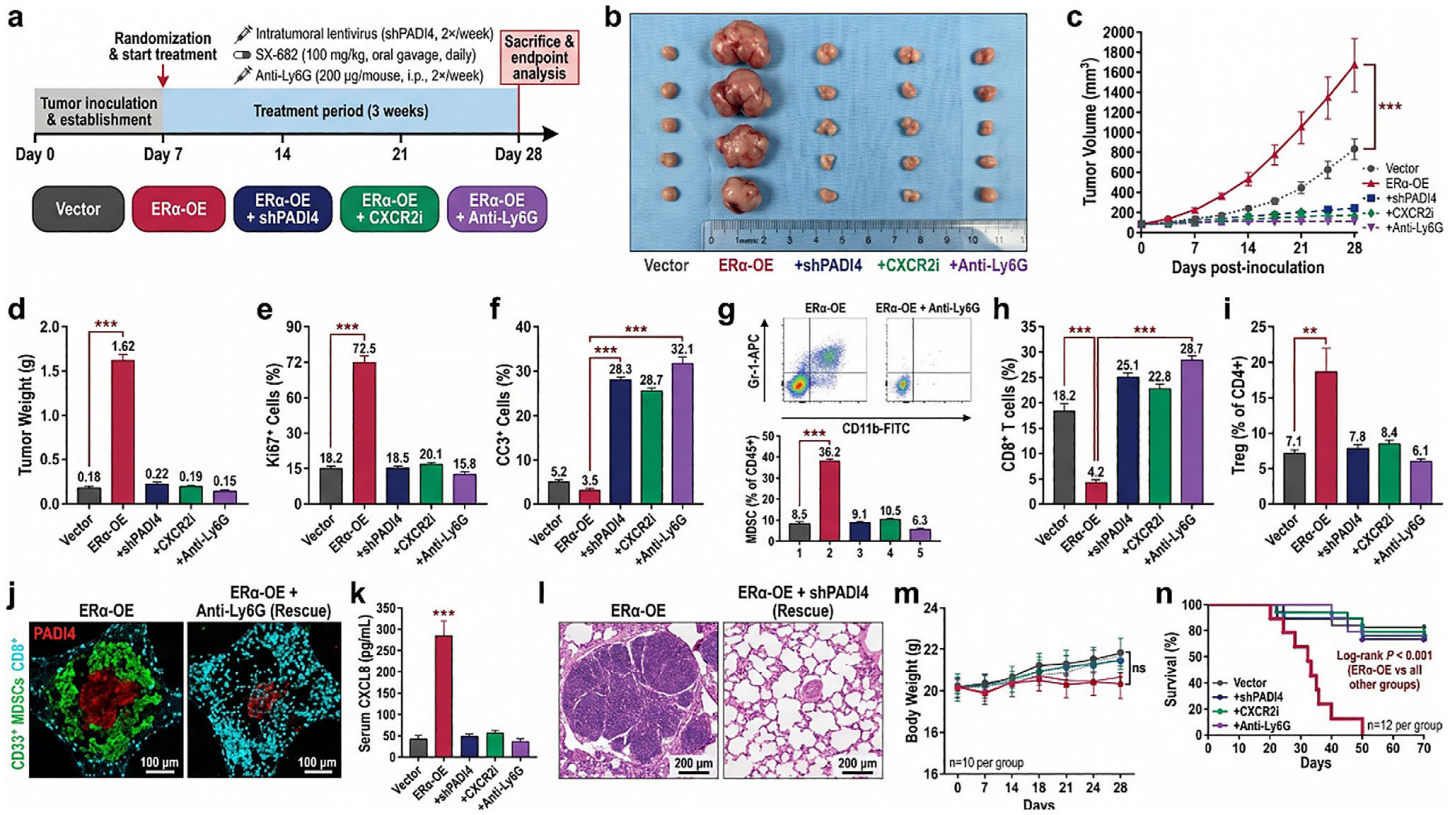
In vivo rescue of the ERα–PADI4–CXCL8–MDSC pathway suppresses tumor growth, remodels the immune microenvironment, and prolongs survival in an orthotopic ATC model. (a) Experimental timeline. 8505C‐ERα‐OE cells (2 × 10^6^) were inoculated subcutaneously on day 0; treatment was initiated on day 7 and continued until sacrifice on day 28. n = 10 per group. (b) Excised tumor photograph at endpoint. Column labels indicate experimental groups. (c) Tumor growth curves. ***p < 0.001, ERα‐OE versus all other groups from day 14 onward. (d) Endpoint tumor weights. ***p < 0.001. (e) Ki67 proliferation index. ***p < 0.001. (f) Cleaved caspase‐3 (CC3) apoptosis index. ***p < 0.001. (g) Flow cytometric quantification of intratumoral MDSCs (CD45^+^CD11b^+^Gr‐1^+^) with representative pseudo‐color plots. ***p < 0.001. (h) Intratumoral CD8^+^ T cell proportion. ***p < 0.001. (i) Intratumoral Treg (CD4^+^FoxP3^+^) proportion. **p < 0.01. (j) Representative multiplex immunofluorescence images of tumor tissues. PADI4 (red), CD33 (green, MDSCs), CD8 (cyan, T cells), DAPI (blue). Scale bar, 100 µm. (k) Serum CXCL8 levels measured by ELISA. ***p < 0.001. (l) Representative H&E images of lung tissues. Arrowheads indicate metastatic nodules. Scale bar, 200 µm. (m) Body weight curves over the treatment period. n.s., not significant. (n) Kaplan–Meier survival curves. Log‐rank *p* < 0.001 for ERα‐OE versus all other groups. Risk table is shown below the x‐axis. For all bar charts and line graphs, data are presented as mean ± SD.

Flow cytometric profiling of the tumor immune microenvironment revealed that ERα‐OE tumors were massively infiltrated by CD11b^+^Gr‐1^+^ MDSCs (38.2 ± 5.1% of CD45^+^ cells), while CD8^+^ T cells were nearly excluded (4.2 ± 1.5%) and FoxP3^+^ regulatory T cells were enriched (18.5 ± 3.2%). All three intervention strategies restored immune surveillance, reducing MDSC proportions to 6%–12%, elevating CD8^+^ T cell frequencies to 22%–30%, and lowering Treg frequencies to 6%–9% (P < 0.001 for each comparison; Figure 5g–i). Multiplex immunofluorescence confirmed that these quantitative changes were accompanied by a spatial remodeling of the tumor microenvironment: the dense CD33^+^ MDSC ring that enveloped PADI4‐high regions in ERα‐OE tumors dissipated, allowing robust infiltration of CD8^+^ T cells into the tumor parenchyma (Figure 5j). Consistent with the reduction in tumor‐intrinsic CXCL8 production, serum CXCL8 levels were five‐ to seven‐fold lower in the rescue groups than in the ERα‐OE group (Figure 5k). Histopathological analysis of lung tissues demonstrated that ERα‐OE tumors gave rise to numerous large, confluent pulmonary metastases, whereas the rescue groups exhibited virtually clean lungs with only rare microscopic foci (Figure 5l). Systemic toxicity was negligible: body weights remained stable across all groups throughout the treatment period (Figure 5m). Histopathological examination of major organs (heart, liver, spleen, lung, kidney, and brain) from all treatment groups revealed normal tissue architecture, with semi‐quantitative scores of 0 for inflammation, necrosis, fibrosis, and vascular changes across all organs examined (Figure S4). The survival benefit was equally pronounced—mice bearing ERα‐OE tumors succumbed by day 50, whereas 70%–85% of mice in the rescue groups survived beyond day 70 (log‐rank P < 0.001; Figure 5n). Collectively, these findings establish that the ERα–PADI4–CXCL8–MDSC axis operates as a functional signaling unit that orchestrates immune evasion and metastatic progression in ATC, and that its blockade—at any of the three examined nodes—is sufficient to restore anti‐tumor immunity and prolong survival.

### 3.6 Combined Pharmacological Blockade of PADI4 and CXCR1/2 Synergistically Suppresses ATC Growth and Restores Anti‐Tumor Immunity

The demonstration that PADI4 inhibition triggers compensatory CXCL1 upregulation via CXCR1/2 (Figure 3k), together with the finding that the residual MDSC chemotaxis observed upon GSK484 treatment can only be fully abrogated by simultaneous blockade of both CXCR1 and CXCR2 (Figure S5), provided a mechanistic rationale for dual targeting of this signaling axis. We therefore evaluated the therapeutic efficacy of combining the PADI4 inhibitor GSK484 with the CXCR1/2 antagonist SX‐682 in the ERα‐OE ATC model (experimental schema, Figure 6a). In vitro ELISA confirmed that GSK484 (10 µM) markedly reduced CXCL8 secretion (795 ± 58 to 72 ± 18 pg/mL, an 88% decline; *p <* 0.001), while simultaneously elevating CXCL1 secretion from 45 ± 12 to 128 ± 28 pg/mL (a 2.8‐fold increase; *p <* 0.05; Figure 6b). In Transwell chemotaxis assays, GSK484 or SX‐682 alone partially reduced MDSC migration (from 395 ± 41 to 158 ± 31 and 122 ± 28 cells/field, respectively), but only the combination of both agents nearly abolished chemotaxis (32 ± 11 cells/field; *p <* 0.001 versus each monotherapy; Figure 6c). In vivo, the combination therapy produced a profound anti‐tumor effect: tumor volume in the Combo group reached only 95 ± 28 mm^3^ at day 28, compared with 1920 ± 240 mm^3^ for Vehicle, 980 ± 135 mm^3^ for GSK484, and 850 ± 128 mm^3^ for SX‐682 (*p <* 0.001; Figure 6d). The combination index calculated by the Chou‐Talalay method was 0.42, confirming strong synergy. Consistent with these volumetric data, Combo‐treated tumors weighed only 0.08 ± 0.03 g versus 1.58 ± 0.21 g for Vehicle (a 95% reduction; *p <* 0.001; Figure 6e), and gross tumor photographs showed that residual nodules in the Combo group were barely visible (Figure 6f). Histological examination of lung tissues revealed numerous large, confluent metastases in Vehicle‐treated mice, whereas the lungs of Combo‐treated mice were virtually free of metastatic lesions (Figure 6g). The survival benefit was equally striking: Vehicle‐treated mice succumbed by day 35, whereas 80% of Combo‐treated mice remained alive at day 80 (log‐rank *p <* 0.001; Figure 6h).

**FIGURE 6:**
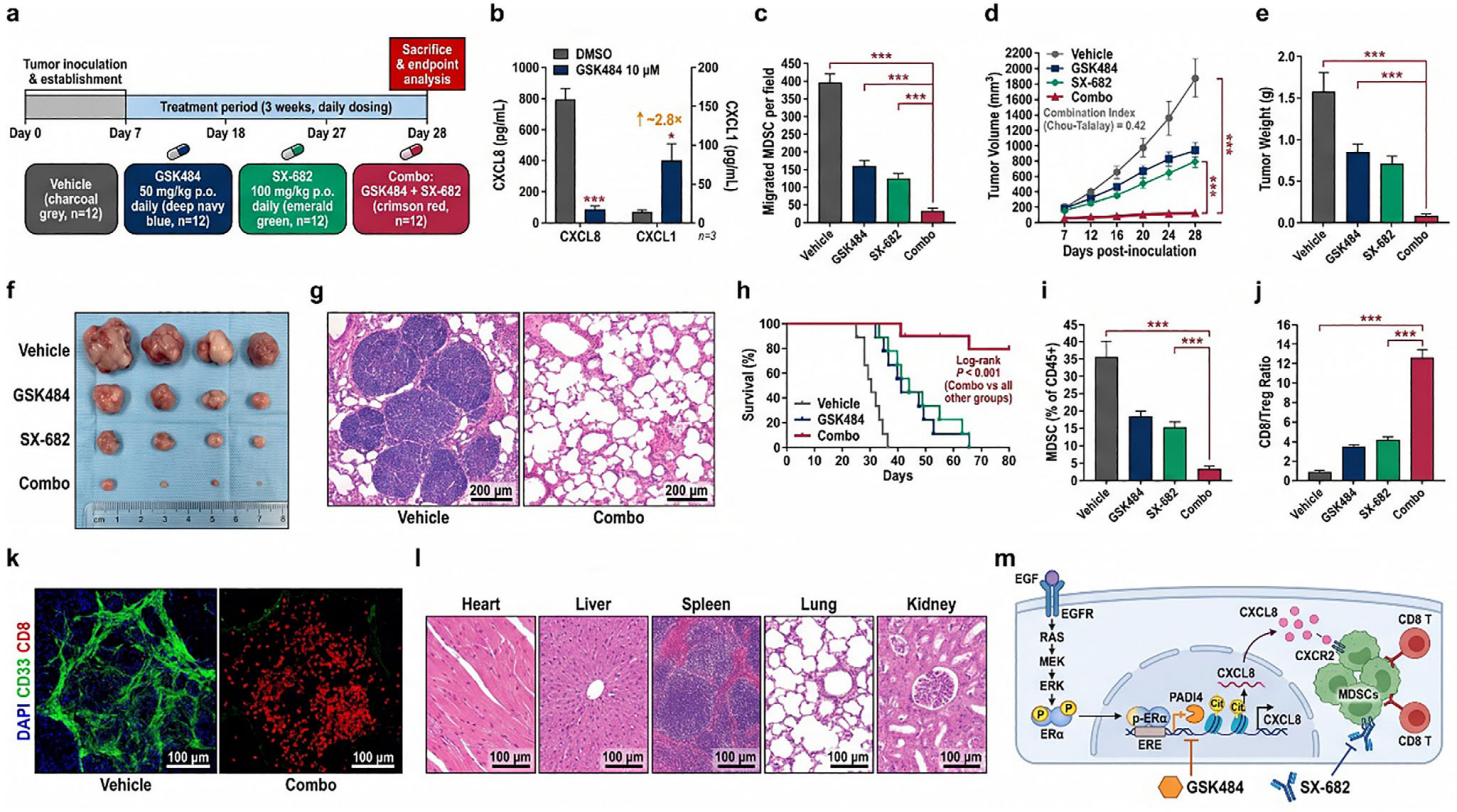
Combined pharmacological inhibition of PADI4 (GSK484) and CXCR1/2 (SX‐682) synergistically suppresses tumor growth, prevents metastasis, and remodels the immune microenvironment in ATC. Experimental timeline for the combination therapy study. 8505C‐ERα‐OE subcutaneous model; daily oral gavage from day 7 to day 27; sacrifice on day 28. n = 12 per group. (b) In vitro compensatory ELISA showing CXCL8 suppression and CXCL1 upregulation upon GSK484 (10 µM) treatment. Left Y‐axis, CXCL8; right Y‐axis, CXCL1. ***p < 0.001, *p < 0.05 versus DMSO. n = 3. (c) In vitro MDSC chemotaxis blocking assay. ***p < 0.001, Combo versus each single agent. n = 5 fields per group. (d) In vivo tumor growth curves. ***p < 0.001, Combo versus all other groups at day 28. Combination Index (Chou‐Talalay) = 0.42. n = 12 per group. (e) Endpoint tumor weights. ***p < 0.001. (f) Excised tumor gross photograph at endpoint. Row labels indicate treatment groups. (g) Representative H&E images of lung tissues. Scale bar, 200 µm. (h) Kaplan‐Meier survival curves. Median survival: Vehicle, 32 days (95% CI: 28–35); GSK484, 52 days (95% CI: 45–58); SX‐682, 55 days (95% CI: 48–62); Combo, not reached (80% survival at Day 80). Log‐rank *p <* 0.001, Combo versus all other groups. Risk table shown below the X‐axis. n = 12 per group. (i) Intratumoral MDSC (CD11b^+^Gr‐1^+^) proportion quantified by flow cytometry. ***p < 0.001. (j) Intratumoral CD8/Treg ratio. ***p < 0.001. (k) Representative multiplex immunofluorescence images of tumor tissues. CD33 (green, MDSCs), CD8 (red, T cells), DAPI (blue, nuclei). Scale bar, 100 µm. (l) Multi‐organ H&E toxicity assessment from the Combo group. Heart, liver, spleen, lung, and kidney sections all show normal histoarchitecture. Scale bar, 100 µm. (m) Master graphical abstract summarizing the ERα–PADI4–CXCL8–MDSC signaling axis and the dual‐intervention strategy with GSK484 and SX‐682. All bar and line graph data are presented as mean ± SD.

Flow cytometric profiling of the tumor immune microenvironment revealed that the Combo regimen dramatically reduced the intratumoral MDSC proportion from 35.5 ± 4.8% (Vehicle) to 3.2 ± 1.1% (*p <* 0.001; Figure 6i), and concurrently elevated the CD8^+^ T cell to Treg ratio from 0.8 ± 0.2 to 12.5 ± 2.8, representing a 15‐fold improvement (*p <* 0.001; Figure 6j). Multiplex immunofluorescence visually confirmed these quantitative shifts: Vehicle tumors exhibited dense CD33^+^ MDSC infiltration with sparse CD8^+^ T cells, whereas Combo tumors showed a near‐complete clearance of MDSCs and robust CD8^+^ T cell infiltration throughout the tumor parenchyma (Figure 6k). Critically, no systemic toxicity was observed with the combination regimen. Histopathological examination of heart, liver, spleen, lung, and kidney tissues from Combo‐treated mice revealed normal tissue architecture without evidence of necrosis, inflammation, or fibrosis (Figure 6l), with semi‐quantitative histopathology scores of 0 for all organs examined (Figure S5), and body weights remained stable throughout the treatment period (Figure 5m). A comprehensive three‐dimensional graphical abstract summarizing the entire signaling cascade—from ligand‐independent ERα activation through PADI4‐mediated CXCL8 secretion to MDSC recruitment and immune evasion, with the dual‐intervention strategy of GSK484 and SX‐682—is presented in Figure 6m.

To confirm that the ERα–PADI4–CXCL8 signaling axis is not unique to 8505C cells, key experiments were repeated in two additional ATC cell lines, CAL‐62 and BHT‐101. In both lines, ERα overexpression significantly transactivated the wild‐type PADI4 promoter, while mutation of the ERE motif completely abolished this activation (Figure S6b,c). PADI4 knockdown in CAL‐62 and BHT‐101 cells significantly reduced CXCL8 secretion with concurrent compensatory CXCL1 upregulation (Figure S6d,e). Furthermore, conditioned medium from sh‐PADI4 CAL‐62 and sh‐PADI4 BHT‐101 cells exhibited markedly reduced MDSC chemotactic activity, which was fully rescued by supplementation with recombinant human CXCL8 (Figure S6f,g). Collectively, these data demonstrate that the ERα–PADI4–CXCL8–MDSC signaling axis operates across multiple ATC cell lines and is not an idiosyncratic feature of 8505C cells. To further validate the prognostic significance of PADI4 in an independent external cohort, we analyzed RNA‐seq data and survival information from the GSE76039 dataset, which includes 18 ATC specimens. Consistent with our single‐center findings, PADI4‐high patients exhibited significantly shorter overall survival (median OS: 4.8 months vs. 15.2 months; HR = 3.15, 95% CI: 1.12–8.85; log‐rank *p =* 0.023; Figure S7), confirming that PADI4 overexpression is a robust adverse prognostic factor in ATC across independent patient populations. Batch‐corrected expression values obtained using the ComBat‐seq algorithm yielded PADI4 and ESR1 expression patterns entirely consistent with the original analysis, confirming that the stepwise increase in PADI4 expression along the dedifferentiation spectrum is not driven by technical batch effects (Figure S8).

## 4 Discussion

In this study, we identified the ERα–PADI4–CXCL8–MDSC signaling axis as a previously unrecognized driver of immune evasion and tumor progression in ATC. Our findings establish a multistep mechanistic framework: ligand‐independent phosphorylation of ERα at S118 by the MAPK pathway directly activates PADI4 transcription; PADI4 citrullinates histone H3 at the CXCL8 promoter to open chromatin and drive CXCL8 secretion; and the secreted CXCL8 recruits MDSCs into the tumor microenvironment through CXCR2, thereby establishing an immunosuppressive niche that excludes CD8^+^ T cells and facilitates metastatic dissemination. Several lines of evidence support the functional integrity and translational relevance of this axis.

The clinical data presented here place PADI4 overexpression at the center of ATC pathobiology. We observed a stepwise increase in PADI4 mRNA and protein levels along the thyroid cancer dedifferentiation spectrum—from normal thyroid to PTC to PDTC to ATC—consistent with a recent spatial transcriptomics study that demonstrated progressive enrichment of MDSC‐related gene signatures and loss of thyroid differentiation markers during malignant evolution from differentiated to anaplastic thyroid cancer [21]. Our findings that PADI4‐high ATC regions are spatially surrounded by dense CD33^+^ MDSC rings with concomitant CD8^+^ T cell exclusion align with the emerging characterization of ATC as a profoundly immunosuppressive malignancy. Recent work has reported that ATC harbors elevated MDSC levels compared with differentiated thyroid cancers, and that these cells are correlated with systemic immunosuppression, enhanced chronic inflammation, and nutritional impairment [22]. Moreover, comprehensive immune profiling has revealed that ATC frequently exhibits a PD‐L1‐positive but CTL‐poor immune microenvironment, a “target‐missing” situation in which MDSCs and Tregs contribute to the limited efficacy of anti‐PD‐1 therapy [23]. The immune‐excluded spatial architecture we documented—with CD33^+^ MDSCs forming a dense annulus around PADI4‐expressing tumor nests while CD8^+^ T cells are restricted to the tumor periphery—provides a mechanistic explanation for these clinical observations and positions PADI4 as an orchestrator of this immune landscape. The finding that PADI4 IHC alone discriminated ATC from PTC with an AUC of 0.92 (95% CI, 0.86–0.98) suggests that PADI4 may serve as a practical diagnostic adjunct in challenging thyroid fine‐needle aspiration or core biopsy specimens, where distinguishing ATC from poorly differentiated or high‐grade PTC can be morphologically ambiguous. However, external validation in independent, multi‐institutional cohorts is required before clinical implementation. We also note that the three sources of normal thyroid tissue used for baseline comparisons—GTEx autopsy specimens for mRNA analysis, fresh‐frozen surgical specimens for protein analysis, and contralateral lobes from tumor‐bearing patients for TMA immunohistochemistry—may exhibit distinct molecular profiles due to differences in tissue collection procedures, ischemic times, and clinical context. However, several lines of evidence suggest that this heterogeneity does not materially affect the conclusions. First, PADI4 expression levels were comparably low across all three normal cohorts, regardless of the source. Second, the stepwise increase in PADI4 expression along the dedifferentiation spectrum observed by mRNA analysis was independently validated by IHC on the TMA cohort, which used contralateral normal thyroid tissue as the comparator. This orthogonal validation, using distinct technical platforms and independent patient cohorts, substantially mitigates concerns about source‐specific bias. Third, the protein‐level findings from fresh‐frozen surgical specimens (Figure 1c) were consistent with the mRNA and IHC data despite the small sample size, further supporting the robustness of the overall conclusion.

The ligand‐independent mechanism we uncovered addresses a long‐standing paradox in thyroid cancer biology: why ATC, which predominantly arises in elderly postmenopausal women with negligible circulating estrogen, exhibits elevated p‐ERα (S118) and ERα transcriptional activity. Our demonstration that EGF‐activated MAPK signaling directly phosphorylates ERα at S118, triggering its nuclear translocation and binding to the PADI4 promoter ERE, extends the paradigm of growth factor‐to‐ERα crosstalk—well‐characterized in breast cancer [9]—to ATC for the first time. This finding has therapeutic implications: it suggests that the ERα–PADI4 axis may remain active even in patients treated with BRAF/MEK inhibitors (dabrafenib plus trametinib), a regimen that has shown significant but short‐lived efficacy in BRAF V600E‐mutant ATC [24]. Therapies targeting the MAPK pathway may inadvertently sustain ERα phosphorylation and PADI4 expression through compensatory signaling, potentially contributing to the immunosuppressive microenvironment that underlies acquired resistance. Recent single‐nucleus RNA‐sequencing of matched primary and resistant ATC tumors has demonstrated that reactivation of the MAPK pathway, accompanied by immunosuppressive myeloid cell expansion, drives acquired resistance to dabrafenib and trametinib [25]. Our work suggests that concurrent targeting of the ERα–PADI4–MDSC axis, in combination with BRAF‐directed therapy, could represent a rational strategy to prevent or delay the emergence of an immunosuppressive resistant state. Notably, the correlation between total ERα and PADI4 IHC scores (Pearson r = 0.68) was stronger than that between p‐ERα(S118) and PADI4 (Pearson r = 0.55). At first glance, this may appear inconsistent with the mechanistic model that phosphorylated ERα, rather than total ERα, drives PADI4 transcription. However, several technical and biological considerations reconcile this apparent discrepancy. First, total ERα IHC is expected to capture both the phosphorylated and unphosphorylated ERα pools, thereby integrating the signal that the phospho‐specific antibody detects only partially. Since total ERα protein abundance is the substrate pool from which p‐ERα is generated, the total ERα signal may serve as a proxy for the cellular capacity to produce p‐ERα upon MAPK pathway activation. Second, phospho‐specific IHC is inherently more technically demanding than total protein IHC. Phosphorylation epitopes are labile and may be partially lost during routine formalin fixation and paraffin embedding due to phosphatase activity prior to complete fixation. Furthermore, the antigen retrieval conditions optimized for total ERα detection (citrate buffer, pH 6.0, microwave heating) may be suboptimal for preserving or exposing the phospho‐S118 epitope, potentially leading to systematic underestimation of the true p‐ERα signal. Third, S118 phosphorylation is a dynamic and potentially substoichiometric modification—at any given moment, only a fraction of total ERα molecules may be phosphorylated at this residue—while total ERα protein levels are relatively stable. Consequently, the total ERα measurement may provide a more time‐integrated and technically robust correlate of ERα transcriptional activity in clinical specimens. Importantly, the functional experiments presented in Figure 4a–i provide orthogonal evidence that is independent of clinical IHC correlations: EGF‐stimulated p‐ERα temporally precedes PADI4 upregulation, MEK inhibition abrogates both p‐ERα and PADI4 induction, ERα knockdown and pharmacological degradation suppress PADI4 expression, and ChIP‐qPCR confirms direct ERα binding to the PADI4 promoter. These complementary lines of evidence collectively establish that phosphorylated ERα, activated through the MAPK pathway, is the functional driver of PADI4 transcription, regardless of the relative strength of clinical IHC correlations.

The downstream mechanism we elucidated—PADI4‐mediated histone H3 citrullination at the CXCL8 promoter—reveals a direct epigenetic link between a chromatin‐modifying enzyme and chemokine‐driven immune evasion. We demonstrated, through a rigorous four‐group ChIP‐qPCR design using wild‐type and catalytically dead (C645S) PADI4 rescue constructs, that the citrullination activity of PADI4 is absolutely required for H3Cit enrichment at the CXCL8 locus, chromatin opening (confirmed by ATAC‐qPCR), and CXCL8 transcriptional activation. This finding extends recent work showing that PADI4 can citrullinate non‐histone substrates—including HIF‐1α at R698 to promote its stabilization in hepatocellular carcinoma [26] and STAT1 at R121 to restrain MHC class II expression in tumor‐associated macrophages [27]—by identifying histone H3 as the key substrate through which PADI4 governs the chemokine secretome of ATC cells. The compensatory upregulation of CXCL1 we observed upon pharmacological PADI4 inhibition with GSK484 is particularly noteworthy. This finding is reminiscent of the adaptive chemokine network rewiring described in other models of targeted therapy resistance, wherein blockade of one chemokine‐receptor axis leads to upregulation of parallel pathways that sustain myeloid cell recruitment [28]. Our demonstration that this compensatory CXCL1 secretion drives residual MDSC chemotaxis through both CXCR1 and CXCR2, and that only dual blockade of both receptors fully abrogated migration, provides a mechanistic rationale for the combination strategy we subsequently tested.

With respect to the pharmacological experiments, we note that GSK484 is the most widely used tool compound for selective PADI4 inhibition, with a reported IC50 of approximately 50 nM for PADI4 in biochemical assays. However, the selectivity profile of GSK484 across the PADI family has not been comprehensively characterized in published studies, and its activity against PADI2 and PADI3—which share considerable structural homology with PADI4 in their catalytic domains—has not been systematically reported. Given that PADI2 has been implicated in macrophage‐mediated immunosuppression, we cannot formally exclude the possibility that some of the observed effects of GSK484—particularly at the 10 µM concentration used in our in vitro experiments—may involve partial inhibition of other PADI family members. Importantly, the convergent results obtained with genetic PADI4 silencing (shRNA) and pharmacological inhibition (GSK484) provide mutual validation: the shRNA experiments demonstrate that selective PADI4 depletion is sufficient to recapitulate the key phenotypes, while the GSK484 experiments confirm that pharmacological targeting of the PADI4 active site achieves comparable effects. This convergence argues against a major contribution of off‐target PADI isoform inhibition to the observed phenotypes. Nonetheless, the development of isoform‐selective PADI inhibitors with well‐characterized selectivity profiles will be important for future preclinical and clinical applications of this therapeutic strategy.

The molecular mechanism underlying the reciprocal regulation of CXCL8 and CXCL1 upon PADI4 inhibition remains to be elucidated, but several plausible models warrant consideration. First, CXCL8 and CXCL1 may share upstream transcriptional regulators—such as NF‐κB, AP‐1, or STAT3—whose recruitment to target gene promoters is differentially sensitive to local chromatin citrullination status. Under basal conditions, PADI4‐mediated histone H3 citrullination at the CXCL8 promoter may favor the binding of transcriptional activators to this locus; upon PADI4 inhibition, reduced citrullination at CXCL8 may release these limiting transcription factors, increasing their availability for binding to the CXCL1 promoter and thereby driving its compensatory upregulation. Second, a chromatin‐level“seesaw” effect may operate between these loci. CXCL8 and CXCL1 are located in close proximity within the CXC chemokine gene cluster on chromosome 4q13.3, separated by approximately 1.5 Mb. Reduced citrullination and chromatin opening at the CXCL8 locus may alter the three‐dimensional chromatin conformation of the broader gene cluster, indirectly affecting the transcriptional competence of neighboring chemokine genes. Third, post‐transcriptional mechanisms may contribute: both CXCL8 and CXCL1 mRNAs contain AU‐rich elements (AREs) in their 3' untranslated regions that regulate mRNA stability through interactions with RNA‐binding proteins. PADI4 may influence the expression or activity of these regulatory factors, thereby exerting differential control over the stability of CXCL8 versus CXCL1 transcripts. Systematic dissection of these possibilities—through transcription factor ChIP‐qPCR, chromosome conformation capture, and mRNA stability assays—represents an important direction for future investigation and may reveal general principles governing adaptive chemokine network rewiring in response to targeted epigenetic disruption.

Our in vivo rescue experiments provide the functional proof that the ERα–PADI4–CXCL8–MDSC axis operates as a cohesive signaling unit in ATC. The observation that silencing PADI4, blocking CXCR2, or depleting MDSCs each independently suppressed tumor growth, reduced pulmonary metastasis, and prolonged survival to a comparable degree indicates that these three nodes function within a linear pathway with limited redundancy. The comparable efficacy of these three distinct interventions supports the concept that PADI4‐driven MDSC recruitment represents a dominant immune evasion mechanism in this model. These findings are consistent with recent work demonstrating that myeloid‐specific deletion of PADI4 reduces the immunosuppressive function of PMN‐MDSCs and enhances T cell‐mediated tumor rejection in syngeneic models [17], and that SX‐682, as a single agent, increases intratumoral CD8^+^ T cell infiltration and inhibits tumor growth in mouse melanoma [29]. However, unlike previous work that focused on PADI4 function within myeloid cells themselves, our study establishes a tumor‐intrinsic role for PADI4 in shaping the chemokine milieu that recruits MDSCs into the TME.

The combination therapy results carry significant translational implications. The strong synergy we observed between GSK484 and SX‐682 (combination index, 0.42), with near‐complete tumor regression and 80% long‐term survival in the Combo group, suggests that simultaneously targeting the upstream enzymatic driver (PADI4) and the downstream compensatory chemokine receptors (CXCR1/2) can achieve durable therapeutic responses in this highly aggressive model. Our strategy parallels recent advances in epigenetic‐immunotherapy combinations: small‐molecule inhibitors targeting epigenetic regulators such as histone deacetylases, DNA methyltransferases, and EZH2 have shown the capacity to reverse immune evasion and sensitize tumors to immune checkpoint blockade [30]. PADI4 inhibitors may represent a novel class of epigenetic immunomodulators that merit further preclinical and clinical investigation, particularly given that GSK484 is a well‐characterized tool compound with established in vivo bioavailability. SX‐682, meanwhile, has completed Phase I evaluation in combination with pembrolizumab in metastatic melanoma and demonstrated a tolerable safety profile with objective responses in heavily pretreated patients who had progressed on anti‐PD‐1 and anti‐CTLA‐4 therapy [29], and is currently being evaluated in Phase II trials for non‐small cell lung cancer and pancreatic adenocarcinoma [31, 32]. Our preclinical data support the evaluation of this combination in ATC, a disease with no effective second‐line options. The multi‐organ histopathological assessment demonstrating no appreciable systemic toxicity at therapeutic doses further supports the safety of this approach, although formal toxicology studies in immunocompetent models are warranted.

Several important limitations of this study should be acknowledged. First, the in vivo experiments were conducted in BALB/c nude mice, which lack a complete adaptive immune system. While this model allowed us to isolate the MDSC‐mediated component of immunosuppression, it precludes assessment of the full interplay between the ERα–PADI4 axis and T cell, B cell, and NK cell compartments. Future studies in syngeneic models with intact immunity—possibly using murine ATC cell lines or genetically engineered mouse models—will be essential to evaluate how this axis interacts with adaptive anti‐tumor immunity and whether the combination of GSK484 and SX‐682 can synergize with immune checkpoint inhibitors. Second, while our multi‐cohort clinical dataset establishes the prognostic significance of PADI4 and the ligand‐independent nature of its regulation, the retrospective design and limited sample size of ATC—an inherently rare disease—constrain the statistical power of subgroup analyses. Prospective validation in larger, multi‐institutional cohorts is necessary before PADI4 can be implemented as a routine clinical biomarker. Third, although we demonstrated that EGF‐activated MAPK signaling drives ERα‐mediated PADI4 transcription, ATC is characterized by a complex mutational landscape including frequent BRAF V600E, RAS, and PI3K‐AKT pathway alterations. Whether distinct oncogenic drivers differentially engage the ERα–PADI4 axis, and whether PADI4 expression is co‐regulated by additional transcription factors—such as NF‐κB, STAT3, or AP‐1, which are also activated in ATC—remains to be determined. Fourth, the CXCL1 compensatory upregulation we observed upon PADI4 inhibition highlights a broader challenge in targeting chemokine networks: tumor cells may exhibit substantial plasticity in their secretome, necessitating combination strategies that anticipate and preempt adaptive rewiring. The molecular mechanism underlying the reciprocal regulation of CXCL8 and CXCL1—whether through shared upstream transcriptional regulators, chromatin‐level compensation, or post‐transcriptional mechanisms—merits dedicated investigation. Fifth, throughout this study, MDSCs were operationally defined as CD11b^+^Gr‐1^+^ cells, a widely used definition that does not discriminate between the monocytic (M‐MDSC: CD11b^+^Ly6G^−^Ly6C^+^) and polymorphonuclear (PMN‐MDSC: CD11b^+^Ly6G^+^Ly6C^−^) subsets. These subsets employ distinct immunosuppressive mechanisms—M‐MDSCs primarily depend on arginase‐1 and inducible nitric oxide synthase, whereas PMN‐MDSCs rely more heavily on reactive oxygen species and NET formation. The relative contribution of each subset to the immunosuppressive phenotype observed in our models remains to be determined. Given that CXCL8‐CXCR2 signaling has been reported to promote the recruitment of both M‐MDSC and PMN‐MDSC populations, it is plausible that the PADI4‐CXCL8 axis influences both subsets, but future studies incorporating Ly6G and Ly6C markers will be necessary to dissect their individual contributions and to determine whether one subset is preferentially affected by the therapeutic interventions tested herein. Sixth, our ATAC‐qPCR analysis was limited to the CXCL8 promoter and a single negative control intergenic region. While the concordance between locus‐specific H3Cit ChIP‐qPCR and ATAC‐qPCR data at the CXCL8 promoter supports a local regulatory mechanism, we cannot formally exclude the possibility that PADI4 knockdown induces broader changes in chromatin accessibility at other genomic loci. The ChIP‐qPCR demonstration that H3Cit enrichment is specifically reduced at the CXCL8 promoter upon PADI4 knockdown—but is restored by wild‐type PADI4 rescue and not by the C645S mutant—provides orthogonal evidence that PADI4‐mediated histone citrullination at this locus is a targeted, enzyme activity‐dependent event. Nonetheless, future genome‐wide studies using ATAC‐seq or ChIP‐seq for H3Cit will be required to comprehensively define the landscape of PADI4‐dependent chromatin remodeling in ATC cells and to determine whether additional chemokine or immunomodulatory genes are regulated through this epigenetic mechanism. Seventh, all in vivo experiments were conducted in BALB/c nude mice, which lack a thymus‐derived T cell compartment. While this model enabled us to isolate the MDSC‐mediated component of immunosuppression and to demonstrate that three mechanistically distinct node‐specific interventions converge on comparable anti‐tumor efficacy, it cannot recapitulate the full interplay between the ERα–PADI4 axis and adaptive immune cell populations—including CD4^+^ T helper cells, CD8^+^ cytotoxic T lymphocytes, B cells, and NK cells. The development of a robust, well‐characterized immunocompetent syngeneic murine ATC model remains an important goal for the field. Such a model would permit comprehensive evaluation of whether GSK484 plus SX‐682 combination therapy reshapes the tumor immune microenvironment in an immunocompetent setting, whether it synergizes with immune checkpoint blockade, and whether durable anti‐tumor immunity involving memory T cell responses can be achieved. These studies are currently being designed in our laboratory and will be pursued as a high priority. Additionally, the PDTC cohort (n = 28) is considerably smaller than the PTC (n = 365) and ATC (n = 65) cohorts, reflecting the inherent rarity of this intermediate‐grade thyroid malignancy. PDTC data are included primarily to illustrate PADI4 expression trends along the dedifferentiation spectrum, and PDTC‐related statistical comparisons should be regarded as exploratory. Larger, multi‐institutional studies with prospective PDTC accrual will be necessary to validate the findings in this specific histological subtype.

Finally, an important conceptual limitation of this study is that it focuses exclusively on tumor‐intrinsic PADI4 and does not dissect the contribution of myeloid‐derived PADI4 to the immunosuppressive phenotype of ATC. PADI4 is expressed in myeloid lineage cells, including MDSCs themselves and tumor‐associated macrophages, and previous work has elegantly demonstrated that myeloid‐specific PADI4 deletion reduces PMN‐MDSC immunosuppressive function and enhances T cell‐mediated tumor rejection in syngeneic models [17]. In the ATC context, it is plausible that tumor‐intrinsic PADI4 and myeloid‐intrinsic PADI4 operate in a cooperative, feed‐forward manner: tumor‐derived PADI4 drives CXCL8 secretion to recruit MDSCs into the tumor bed, while myeloid‐derived PADI4 sustains the immunosuppressive function of these recruited cells. The relative contributions of these two cellular sources, and the extent to which they are therapeutically targetable by systemic PADI4 inhibition, remain to be determined. Future studies employing bone marrow chimeras or conditional PADI4 knockout models (e.g., PADI4^fl/fl^ crossed with Lyz2‐Cre for myeloid‐specific deletion) will be essential to genetically disentangle these distinct roles.

Beyond the specific therapeutic strategy evaluated here, our findings raise broader questions about the role of protein citrullination in tumor immunity. PADI4 is one of five members of the PADI family, and while PADI2 has been implicated in macrophage‐mediated immunosuppression, the potential for functional redundancy or compensation among PADI family members in the TME is largely unexplored. Furthermore, citrullinated proteins can serve as neoantigens, and anti‐citrullinated protein antibodies are well‐established biomarkers in rheumatoid arthritis. Whether PADI4 activity in ATC generates citrullinated neoepitopes that could be exploited for cancer vaccination or adoptive T cell therapy represents an intriguing direction for future research. The recent discovery that dihydroergotamine mesylate acts as a PADI4 antagonist and suppresses tumor progression [26] provides an additional pharmacological tool for probing these questions.

In conclusion, this study identifies the ERα–PADI4–CXCL8–MDSC signaling axis as a central driver of immune evasion in ATC, delineates the upstream and downstream regulatory mechanisms with genetic, pharmacological, and clinical evidence, and establishes proof‐of‐concept for a dual‐targeted therapeutic strategy. These findings expand our understanding of how oncogenic kinase signaling is coupled to epigenetic regulation of the tumor immune microenvironment through non‐canonical ERα activation, and suggest that pharmacological disruption of this axis—at the level of PADI4, CXCR1/2, or both—merits clinical investigation in this disease of critical unmet need.

## Author Contributions

Dongdong Zhang contributed to the design of the study protocol. Ying Cai and Junbin Zhang performed the statistical analysis. Dongdong Zhang, Junbin Zhang, and Jianyong Zhang prepared the figures. Ran Jing contributed to the writing of the study protocol and made the final corrections to th is manuscript. Ming Cai finalized the manuscript. All authors read and approved the final manuscript.

## Funding

National Natural Science Foundation of China Under Grand 82502837.

## Ethics Statement

The clinical study was approved by the Ethics Committee of Chongqing University Cancer Hospital, and written informed consent was obtained from all patients. All animal experiments were approved by the Institutional Animal Care and Use Committee (IACUC) of Chongqing University Cancer Hospital and performed in accordance with institutional guidelines.

## Conflicts of Interest

The authors declare that the research was conducted in the absence of any commercial or financial relationships that could be construed as a potential conflicts of interest.

## Supporting information




**Supporting File**: advs77901‐sup‐0001‐SuppMat.docx.

## Data Availability

The RNA‐seq data generated in this study are being deposited in the Gene Expression Omnibus (GEO) and the accession number will be provided upon completion of the submission. In the interim, the data are available from the corresponding author upon reasonable request. All other data generated or analyzed during this study are included in the article and its supplementary information files.
