# Supplementary material for: ERα–PADI4–CXCL8 Axis Drives MDSC‐Mediated Immune Evasion in Anaplastic Thyroid Carcinoma
Source: Adv Sci (Weinh). 2026 Oct 22:e77901. Online ahead of print. doi: 10.1002/advs.77901 (PMC13691237; doi:10.1002/advs.77901)
Supplement: Supplementary file 1 [file ADVS-9999-e77901-s001.docx]

**Supplement Figure Legend**

**Supplement Figure S1. Baseline clinicopathological characteristics of the ATC cohort stratified by PADI4 expression.**
Data are presented as mean ± SD, median (IQR), or n (%). PADI4-Low and PADI4-High groups are defined by median PADI4 IHC H-score (cutoff = 120). P-values were calculated using unpaired Student's t-test for continuous variables, Mann-Whitney U test for TSH, and Chi-squared or Fisher's exact test for categorical variables. *P < 0.05, **P < 0.01, ***P < 0.001. Abbreviations: TSH, thyroid-stimulating hormone; IQR, interquartile range; AJCC, American Joint Committee on Cancer; R0/R1, microscopically margin-negative/positive resection; R2, macroscopically incomplete resection; TERT, telomerase reverse transcriptase.

**Supplement Figure 2**. **Global transcriptomic profiling of PADI4-depleted ATC cells reveals dual regulation of immune chemotactic and apoptotic pathways**

RNA-sequencing heatmap of 8505C ATC cells stably transduced with non-targeting control (sh-NC) or sh-PADI4 lentivirus (n = 3 independent biological replicates per group). Libraries were sequenced on an Illumina NovaSeq 6000 platform (150 bp paired-end), reads were aligned to GRCh38 using STAR, and gene-level counts were quantified with featureCounts. Differential expression analysis was performed with DESeq2 using thresholds of |log_2_ fold change| > 1.0 and adjusted P-value (Benjamini-Hochberg) < 0.05. A total of 1,250 differentially expressed genes were identified (680 upregulated, 570 downregulated). The heatmap displays the top 40 upregulated and top 40 downregulated genes (80 rows total), with each column representing an individual biological replicate. Data are presented as row Z-scores of regularized log-transformed (rlog) counts. Hierarchical clustering was performed using Euclidean distance and complete linkage. Column dendrograms confirm perfect separation of sh-NC and sh-PADI4 replicates. Key genes are annotated with functional category indicators: chemokines (orange dots), cytokines/growth factors (green dots), ECM/invasion-related factors (brown dots), and apoptosis/stress-response genes (purple dots). Note that *CXCL8, CXCL1, CXCL2, CXCL5, CCL20, CSF2, IL6*, and *MMP9* are among the most prominently downregulated genes in sh-PADI4 cells, whereas *CDKN1A (p21)*, *GADD45B, DDIT3 (CHOP), BBC3 (PUMA)*, and FAS are among the most prominently upregulated genes. These results identify PADI4 as a dual-function transcriptional regulator that simultaneously promotes an immunosuppressive secretome while suppressing apoptotic and stress-response programs in ATC cells, providing transcriptome-wide mechanistic support for the phenotypic observations.

**Supplement Figure S3. Co-localization of ERα and PADI4 in the nucleus of ATC cells upon EGF stimulation.**
Representative confocal dual-immunofluorescence images of 8505C ATC cells serum-starved for 16 h and stimulated with EGF (50 ng/mL, 30 min) to induce ERα nuclear translocation. Cells were co-stained with anti-ERα (rabbit monoclonal, CST #8644) followed by anti-rabbit IgG-Alexa Fluor 488 (green), anti-PADI4 (mouse monoclonal, Abcam ab214810) followed by anti-mouse IgG-Alexa Fluor 555 (red), and mounted with ProLong Gold containing DAPI (blue). Images were acquired on a confocal laser scanning microscope with a 63× oil immersion objective using sequential channel acquisition. (Top-left) DAPI staining reveals the large, irregular, pleomorphic nuclei characteristic of ATC cells. (Top-right) ERα (green) is predominantly localized within the nuclear compartment after EGF stimulation. (Bottom-left) PADI4 (red) displays a punctate nuclear and perinuclear staining pattern consistent with its function as a chromatin-modifying enzyme. (Bottom-right) In the merged image, areas of red-green overlap appear as yellow/orange foci (representative foci indicated by white arrows), demonstrating spatial co-localization of ERα and PADI4 within the nucleus. Scale bars, 10 μm. These data provide cytological evidence that ERα and PADI4 occupy the same subnuclear compartment, supporting the ChIP-qPCR finding that ERα directly binds the PADI4 promoter.

**Supplement Figure S4**. **Multi-organ histopathological toxicity assessment of the GSK484 plus SX-682 combination regimen, complementing the five-organ assessment shown in Fig. 6l.**

Representative brightfield H&E-stained sections of heart, liver, spleen, lung, kidney, and brain from BALB/c nude mice bearing 8505C-ERα-OE xenografts treated with GSK484 (50 mg/kg/day, p.o.) and SX-682 (100 mg/kg/day, p.o.) for three weeks (Day 7 to Day 28). Tissues were fixed in 10% neutral buffered formalin, paraffin-embedded, sectioned at 5 µm, and stained with hematoxylin and eosin. All images were acquired at 200× total magnification (scale bar, 100 μm). Histopathological evaluation was performed in a blinded manner by a board-certified veterinary pathologist using a semi-quantitative scoring system (0 = no lesion, 1 = minimal, 2 = mild, 3 = moderate, 4 = severe) for inflammation, necrosis, fibrosis, and vascular changes. All six organs examined displayed normal histoarchitecture with histopathology scores of 0 (no significant lesions) in all categories (n = 6 mice per group). These results, together with the stable body weight data shown in Fig. 5m, demonstrate that the GSK484 + SX-682 combination therapy does not cause appreciable systemic toxicity in this preclinical model.

**Supplement Figure S5**. **Compensatory CXCL1-driven residual MDSC chemotaxis upon GSK484 treatment is mediated through both CXCR1 and CXCR2, and is fully abrogated only by dual receptor blockade.**

(a) Representative brightfield Transwell images of MDSC chemotaxis toward conditioned medium (CM) from 8505C ATC cells treated with DMSO (Vehicle) or GSK484 (10 μM, 24 h). Where indicated, MDSCs were pre-incubated for 30 min with anti-CXCR2 neutralizing antibody (clone 48311, 10 μg/mL), anti-CXCR1 neutralizing antibody (clone 42705, 10 μg/mL), or both antibodies combined prior to seeding. Scale bar, 50 μm. (b) Quantification of migrated MDSCs per field across the five experimental conditions. Data are presented as mean ± SD; n = 5 random fields per group. ***P < 0.001, *P < 0.05, ns = not significant (one-way ANOVA with Tukey post-hoc test). Groups: Vehicle CM (charcoal grey), GSK484 CM (deep navy blue), GSK484 CM + anti-CXCR2 (light blue), GSK484 CM + anti-CXCR1 (pale blue), GSK484 CM + anti-CXCR1/2 (crimson red). GSK484 treatment alone reduced MDSC chemotaxis by approximately 59% relative to Vehicle (from ~412 to ~168 cells/field; P < 0.001). Individual blockade of CXCR2 or CXCR1 further reduced migration to ~82 and ~105 cells/field, respectively (P < 0.001 and P < 0.05 versus GSK484 alone). Critically, dual blockade of both receptors (mimicking the pharmacological action of SX-682) reduced migration to ~35 cells/field (P < 0.001 versus each single antibody), nearly eliminating residual chemotaxis. These data demonstrate that the compensatory CXCL1 upregulation observed upon PADI4 inhibition (see Fig. 3k) drives residual MDSC recruitment through both CXCR1 and CXCR2, providing a mechanistic basis for the combination therapy strategy employing GSK484 and SX-682.

**Supplement Figure S6. Validation of the ERα–PADI4–CXCL8 signaling axis in additional ATC cell lines.**

(a) Schematic overview. (b,c) Dual-luciferase reporter assays in CAL-62 (b) and BHT-101 (c). (d,e) CXCL8 and CXCL1 ELISA in CAL-62 (d) and BHT-101 (e). (f,g) MDSC chemotaxis with rCXCL8 rescue in CAL-62 (f) and BHT-101 (g). Data are mean ± SD; n = 3. ***P < 0.001, **P < 0.01; ns, not significant.

**Supplement Figure S7. External validation of the prognostic significance of PADI4 in ATC.**

Kaplan-Meier overall survival curves for ATC patients from the GSE76039 cohort (n = 18), stratified by median PADI4 mRNA expression. PADI4-high patients had significantly shorter overall survival (median OS: 4.8 months vs. 15.2 months; HR = 3.15, 95% CI: 1.12–8.85; log-rank P = 0.023).

**Supplement Figure S8. Validation of PADI4 and ESR1 expression patterns after ComBat-seq batch correction.**

(a) Box plot of PADI4 mRNA expression after ComBat-seq batch effect correction. ATC retains the highest expression. ***P < 0.001 vs. all other groups; **P < 0.01 for ATC vs. PDTC. (b) Box plot of ESR1 mRNA expression after batch correction, showing no significant difference among groups (n.s., P = 0.38). Data are from GTEx (Normal), TCGA (PTC), and in-house (PDTC/ATC) cohorts.

**Supplementary Table 1: Multivariate linear regression analysis of PADI4 expression with adjustment for clinical confounders.**

| **Variable** | **β Coefficient** | **Standard Error** | **95% CI** | **t-value** | **P-value** |
| --- | --- | --- | --- | --- | --- |
| **Histological group** | | | | | |
| Normal (reference) | — | — | — | — | — |
| PTC | 2.12 | 0.18 | 1.77 to 2.47 | 11.78 | < 0.001 |
| PDTC | 3.78 | 0.28 | 3.23 to 4.33 | 13.50 | < 0.001 |
| ATC | 5.62 | 0.39 | 4.85 to 6.39 | 14.41 | < 0.001 |
| **Age (continuous, per year)** | 0.008 | 0.006 | −0.004 to 0.020 | 1.33 | 0.18 |
| **Sex** | | | | | |
| Female (reference) | — | — | — | — | — |
| Male | 0.15 | 0.12 | −0.09 to 0.39 | 1.25 | 0.21 |
| **Data source** | | | | | |
| GTEx (reference) | — | — | — | — | — |
| TCGA | 0.22 | 0.15 | −0.07 to 0.51 | 1.47 | 0.14 |
| In-house | 0.18 | 0.16 | −0.13 to 0.49 | 1.13 | 0.26 |

PADI4 expression (log_2_ TPM, batch-corrected by ComBat-seq) was set as the dependent variable. Histological group (Normal, PTC, PDTC, ATC), age (continuous), sex (Female, Male), and data source (GTEx, TCGA, In-house) were included as independent variables. The overall model was statistically significant (F = 112.4, P < 0.001; adjusted R^2^ = 0.58). After adjustment for all covariates, histological group remained the dominant determinant of PADI4 expression, with β for ATC vs. Normal = 5.62 (95% CI: 4.85–6.39, P < 0.001). Age, sex, and data source were not significantly associated with PADI4 expression after adjustment (all P > 0.05). CI, confidence interval.

**Supplementary Table 2: Division Index of CD8⁺ T cells in CFSE proliferation assays.**

| **Experimental Condition** | **Division Index (Mean ± SD)** | **P-value (vs. T + sh-NC MDSC)** |
| --- | --- | --- |
| T cells alone | 2.45 ± 0.31 | < 0.001 |
| T cells + sh-NC MDSC | 0.18 ± 0.06 | — |
| T cells + sh-PADI4 MDSC | 1.89 ± 0.28 | < 0.001 |

P-values were calculated by one-way ANOVA with Tukey post-hoc test. Division Index was calculated using the Proliferation Modeling module of FlowJo software (v10.8.1).

**Supplementary Table 3: Quantification of ERα and PADI4 nuclear co-localization in EGF-stimulated 8505C cells.**

| **Parameter** | **Mean ± SD** | **n (cells)** | **n (experiments)** |
| --- | --- | --- | --- |
| Pearson's correlation coefficient | 0.62 ± 0.11 | ≥30 | 3 |
| Manders' overlap coefficient (PADI4 overlapping with ERα) | 0.71 ± 0.09 | ≥30 | 3 |
